# Multiple classes and isoforms of the RNA polymerase recycling motor protein HelD

**DOI:** 10.1002/mbo3.1251

**Published:** 2021-12-08

**Authors:** Joachim S. Larsen, Michael Miller, Aaron J. Oakley, Nicholas E. Dixon, Peter J. Lewis

**Affiliations:** ^1^ School of Environmental and Life Sciences University of Newcastle Callaghan New South Wales Australia; ^2^ School of Chemistry and Molecular Bioscience University of Wollongong and Illawarra Health and Medical Research Institute Wollongong New South Wales Australia

**Keywords:** gene expression regulation, helicases, phylogenetic analysis, RNA polymerase

## Abstract

Efficient control of transcription is essential in all organisms. In bacteria, where DNA replication and transcription occur simultaneously, the replication machinery is at risk of colliding with highly abundant transcription complexes. This can be exacerbated by the fact that transcription complexes pause frequently. When pauses are long‐lasting, the stalled complexes must be removed to prevent collisions with either another transcription complex or the replication machinery. HelD is a protein that represents a new class of ATP‐dependent motor proteins distantly related to helicases. It was first identified in the model Gram‐positive bacterium *Bacillus subtilis* and is involved in removing and recycling stalled transcription complexes. To date, two classes of HelD have been identified: one in the low G+C and the other in the high G+C Gram‐positive bacteria. In this work, we have undertaken the first comprehensive investigation of the phylogenetic diversity of HelD proteins. We show that genes in certain bacterial classes have been inherited by horizontal gene transfer, many organisms contain multiple expressed isoforms of HelD, some of which are associated with antibiotic resistance, and that there is a third class of HelD protein found in Gram‐negative bacteria. In summary, HelD proteins represent an important new class of transcription factors associated with genome maintenance and antibiotic resistance that are conserved across the Eubacterial kingdom.

## Graphical abstract

Stalled transcription complexes must be removed to prevent harmful collisions with DNA replication and actively transcribing complexes. This work investigates the phylogeny of recently identified HelD proteins that play an important role in removing stalled complexes. Transcription recycling factor HelD is widely distributed across the eubacteria as is represented by three different classes: Class I in the low G+C Gram‐positives; Class II in the high G+C Gram‐positives; Class III in the Gram‐negative deltaproteobacteria. Many organisms contain multiple expressed isoforms of HelD.

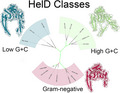

## 1 INTRODUCTION

Transcription elongation is punctuated by pauses that serve important functions in permitting correct folding of structural RNA, efficient coupling of transcription and translation, and ensuring efficient transcription termination at the correct site (Saba et al., 2019). Whilst most pausing events serve an important function, on occasion RNA polymerase (RNAP) is unable to restart transcription and must be removed from the DNA to prevent damaging collisions with the DNA replication machinery or other transcription complexes (Adelman & Lis, 2012; Gupta et al., 2013; Pomerantz & O'Donnell, 2008, 2010, Rocha, 2004). Several systems used to resolve stalled transcription complexes have been characterized; for example, Mfd has been shown to bind to stalled transcription complexes (either a stochastic pause during transcription of structured RNA or at a site of DNA damage), physically removing it from the DNA or restarting it *via* a RecG‐like ATPase motor domain (Ghodke et al., 2020; Ho et al., 2018; Kang et al., 2021; Le et al., 2018; Ragheb et al., 2021; Shi et al., 2020; Westblade et al., 2010). In *B*. *subtilis* RNaseJ1 clears stalled RNAP using a torpedo mechanism (5′–3′ exonuclease activity followed by RNAP displacement) (Sikova et al., 2020), and in *Escherichia coli* the helicase protein RapA is important in recycling RNAP (Liu et al., 2015). UvrD/PcrA in concert with Gre factors has been reported to act on RNAP stalled at a DNA lesion, binding to the complex and using the energy of ATP hydrolysis to backtrack away from the lesion to allow repair systems access to the damaged DNA (Epshtein et al., 2014; Hawkins et al., 2019), although it now appears that the role of these helicases is in preventing the formation of, and resolving, R‐loops (RNA‐DNA hybrids) that can have a detrimental effect on DNA replication (Urrutia‐Irazabal et al., 2021).

**FIGURE 1:**
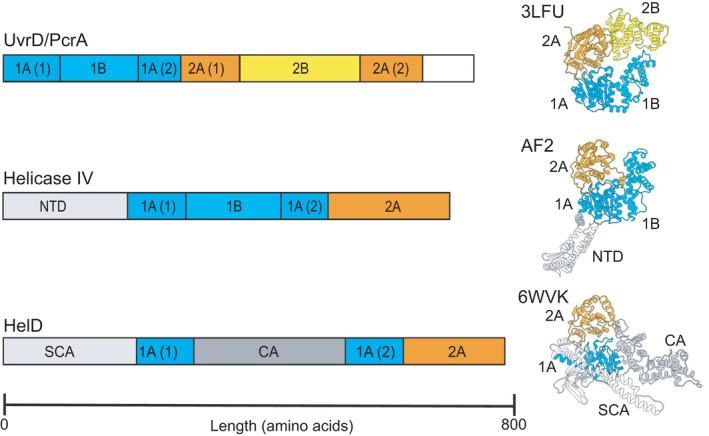
Relationship between UvrD/PcrA and helicase IV/HelD proteins. The left side shows scaled linear representations of the domain organization of superfamily 1 (SF1) helicase UvrD/PcrA (top), *Escherichia coli* helicase IV (middle), and *B*. *subtilis* HelD (bottom). A scale bar (amino acids) is shown at the bottom. The right‐hand side shows structures, aligned *via* their 1A and 2A domains, with domains colored corresponding to the left panels. Top, UvrD (PDB ID 3LFU); middle, helicase IV (AlphaFold2 model, AF2); bottom, HelD (taken from RNAP‐HelD complex PDB ID 6WVK). 1A, B, 2A, and 2B refer to conserved SF1 helicase domains. NTD, SCA, and CA refer to the AlphaFold2 modeled N‐terminal domain of helicase IV and the secondary channel arm and clamp arm of HelD, respectively

An additional system identified in Gram‐positive bacteria required for recycling stalled transcription complexes involves the action of the motor protein HelD (Wiedermannova et al., 2014). The designation of HelD (also called helicase IV) was originally made for a protein identified in *E*. *coli* as a weakly processive 3′–5′ DNA helicase (Wood & Matson, 1987). To avoid confusion with the separate classes of HelD proteins that are the focus of this work, the *E*. *coli* protein will be referred to as helicase IV. Based on conserved sequence motifs Helicase IV is a superfamily 1 (SF1) helicase, related to housekeeping helicase UvrD/PcrA (Figure 1). The *B*. *subtilis* gene *yvgS* was assigned the name *helD* based on limited protein sequence conservation to helicase IV (Wiedermannova et al., 2014), although the proteins differed with respect to domain organization (Koval et al., 2019; Wiedermannova et al., 2014) (Figure 1). Little functional, and no structural information is available for helicase IV, although a model generated by AlphaFold2 (Jumper et al., 2021) enables tentative comparison of UvrD/PcrA, helicase IV, and *B*. *subtilis* HelD (Figure 1). Helicase IV and HelD show similarity with UvrD/PcrA around the well‐defined 1A and 2A helicase domains (blue and orange, respectively, Figure 1a), but not in other structural motifs associated with helicase activity (UvrD/PcrA domains 1B and 2B). Both helicase IV and HelD have N‐terminal domains not present in UvrD/PcrA helicases, and helicase IV has a putative 1B domain which may account for its reported helicase activity, whilst in the equivalent 1B domain position HelD contains an unrelated sequence that folds into a novel clamp‐arm (CA) structure important in transcription recycling (Newing et al., 2020; Wiedermannova et al., 2014). Whilst UvrD/PcrA and helicase IV have helicase activity, HelD shows none suggesting it has evolved from an SF1‐type helicase into a transcription recycling factor that utilises the energy from ATP hydrolysis catalysed by its helicase motifs for its transcription‐related activity.

Studies on HelD from low G+C (*Bacillus subtilis*) and high G+C (*Mycobacterium smegmatis*) Gram‐positives revealed that there are two distinct classes of the enzyme, confirmed by phylogenetic and structural analyses (Kouba et al., 2020; Newing et al., 2020; Pei et al., 2020). Class I HelD was described from *B*. *subtilis*, whilst the structurally distinct Class II enzyme was identified in *M*. *smegmatis* (Kouba et al., 2020; Newing et al., 2020; Pei et al., 2020). Class I and II HelDs have similar motor domains but differ in the structure of their arms and the mechanism by which these arms perform the mechanical activity of removing nucleic acids and recycling RNAP (Kouba et al., 2020; Newing et al., 2020; Pei et al., 2020).

**FIGURE 2:**
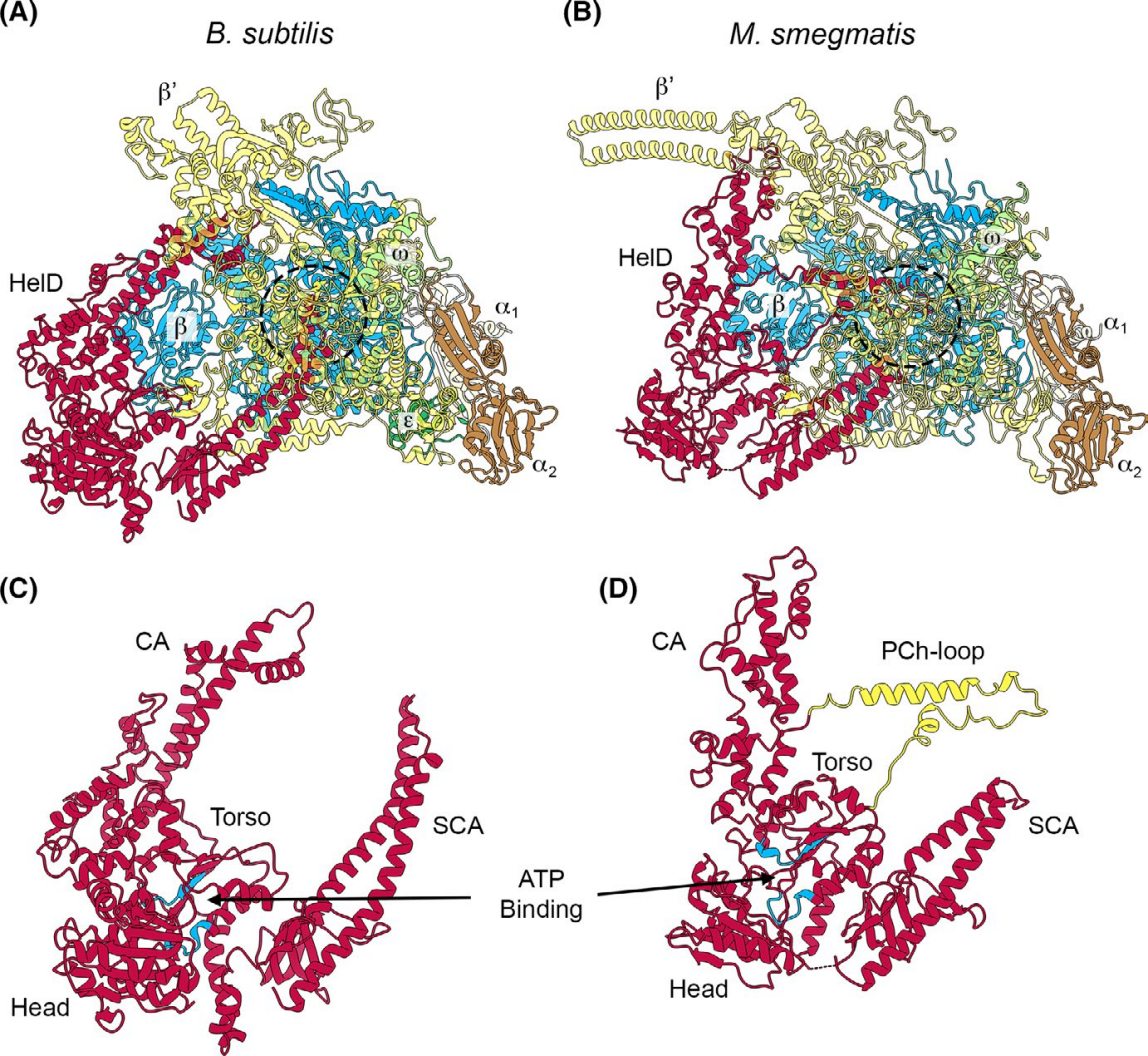
The two known structural classes of HelD. Panel A shows the structure of the *B*. *subtilis* RNAP‐Class I HelD complex (PDB ID 6WVK). Panel B shows the *M*. *smegmatis* RNAP‐Class II HelD complex (PDB ID 6YYS; state II). RNAP subunits and HelDs are colored identically in both panels with the transparency of the β’ subunit set at 50% so that HelD structures adjacent to the RNAP active site region (dashed circles) can be more easily visualized. Panels C and D show HelD structures in the same orientation as in Panels A and B, with the ATP binding site colored in blue and the PCh‐loop from *M*. *smegmatis* HelD colored in yellow (see text for details)

The recent structures of HelD from *B*. *subtilis* and *M*. *smegmatis* bound to core RNAP (α_2_ββ’ω) (Kouba et al., 2020; Newing et al., 2020) are shown in Figure 2a and b, along with the Class I *B*. *subtilis* (Figure 2c) and Class II *M*. *smegmatis* (Figure 2d) enzymes. HelD has an unusual mode of action dependent on two arms (CA and SCA, Figure 2c and d) attached to the central UvrD‐like ATPase motor domain (Head and Torso, Figure 2c and d), in which nucleic acids are pushed out of the active site whilst the DNA binding clamp and RNA exit channels are simultaneously opened, leading to the release of the stalled RNAP (Newing et al., 2020). This recycling activity is powered by ATP hydrolysis and the mechanical action of the two arms that flank the motor domain. In the Class I HelD, the long SCA (Figure 2a and c) can physically remove nucleic acids from the active site (dotted circle in Figure 2a), whereas in the Class II HelD the SCA is too short, and instead nucleic acid removal is performed by a CA insert called the PCh‐loop (Figure 2b and d) (Kouba et al., 2020; Newing et al., 2020). Recent reports also suggest that some Class II HelDs (from *M*. *abscessus* and *Streptomyces venezuelae*) can confer rifampicin resistance through removal of rifampicin by the PCh‐loop (Hurst‐Hess et al., 2021; Surette et al., 2021).

In this work, we take advantage of the recent structural information to compile a detailed phylogenetic analysis of HelD showing that many organisms contain more than one (up to 5) different versions of HelD, that the genes encoding these enzymes are all expressed, that HelD is likely to have been acquired by horizontal gene transfer in Gram‐negative *Bacteroides* and Gram‐positive *Coriobacteria* and *Acidimicrobiia*, and that there is a third Class of HelD found in the Gram‐negative *Deltaproteobacteria*.

## 2 EXPERIMENTAL PROCEDURES

### 2.1 Sequence retrieval and analysis

The sequence of *B*. *subtilis* 168 HelD (UniProtKB ID: O32215) was used to search for homologues on 11/11/2020 using the NCBI Conserved Domain Architecture Retrieval Tool (Geer et al., 2002), which identified 13,781 sequences, which were trimmed to 11,821 to remove partial sequences (<600 aa). To aid subsequent analyses, particularly for the study of multiple copies of *helD* genes, the original sequences were used to search complete reference genomes from the KEGG (https://www.kegg.jp) and JGI (https://jgi.doe.gov) databases. HelD and RpoB sequences retrieved from these complete genomes were used for subsequent phylogenetic studies.

### 2.2 Construction of phylogenetic trees

Selected sequences were aligned using MAFFT (Katoh et al., 2002, 2019) with default settings. Sequence alignments were then trimmed using Gblocks (https://ngphylogeny.fr). The best‐fitting model (LG) was determined using ProtTest 3 (Darriba et al., 2011) and phylogenetic trees were constructed using MrBayes 3.2 (Huelsenbeck & Ronquist, 2001; Ronquist et al., 2012), which were run until the standard deviation was below 0.01. Phylogenetic trees were also made on MEGA‐X (Kumar et al., 2018), using the Maximum Likelihood statistical method with 1000 bootstrap replications, and using RAxML (Stamatakis, 2006) using default settings. All trees had the same topology. Trees were visualised using iTol (Letunic & Bork, 2019).

### 2.3 Transcriptome data and analysis

Gene expression data were obtained from datasets deposited in the Sequence Read Archive (SRA; https://www.ncbi.nlm.nih.gov/sra) and were: *B*. *subtilis* 168 (Revilla‐Guarinos et al., 2020); *B*. *cereus* F837/76 (Jessberger et al., 2019); *Clostridium perfringens* 13 (Soncini et al., 2020); *Streptomyces coelicolor* A3(2) (Jeong et al., 2016); *Mycobacterium smegmatis* MC2‐155 (Feng et al., 2020); *Myxococcus xanthus* DK1622 (SRA accession code: PRJNA516475); *Bacteroides vulgatus* ATCC8482 (SRA accession code: PRJNA473003). Reads were mapped to the respective reference genome sequences, and gene expression levels were calculated in Genious Prime 2020.2.3 (https://www.geneious.com). Transcript per million (TPM) values were used for comparison of *helD* expression levels *cf*. *rpoB*, and *pcrA*/*uvrD* (for *S*. *coelicolor* A3(2)).

### 2.4 Structure modeling

RNAP RpoB (β) and RpoC (β’) subunits from *M*.* xanthus* DK1622 were modeled in SWISS‐MODEL (Waterhouse et al., 2018) using *E*. *coli* RNAP, PDB ID: 6ALF (Kang et al., 2017) as a defined template. The *M*.* xanthus* HelD structure was modeled using i‐Tasser (Yang et al., 2015) with output model 1 (C‐score −0.48) selected for presentation in this work. Structural images used in this work were prepared in ChimeraX (Pettersen et al., 2021).

## 3 RESULTS AND DISCUSSION

### 3.1 Distribution and phylogeny of HelD

**FIGURE 3:**
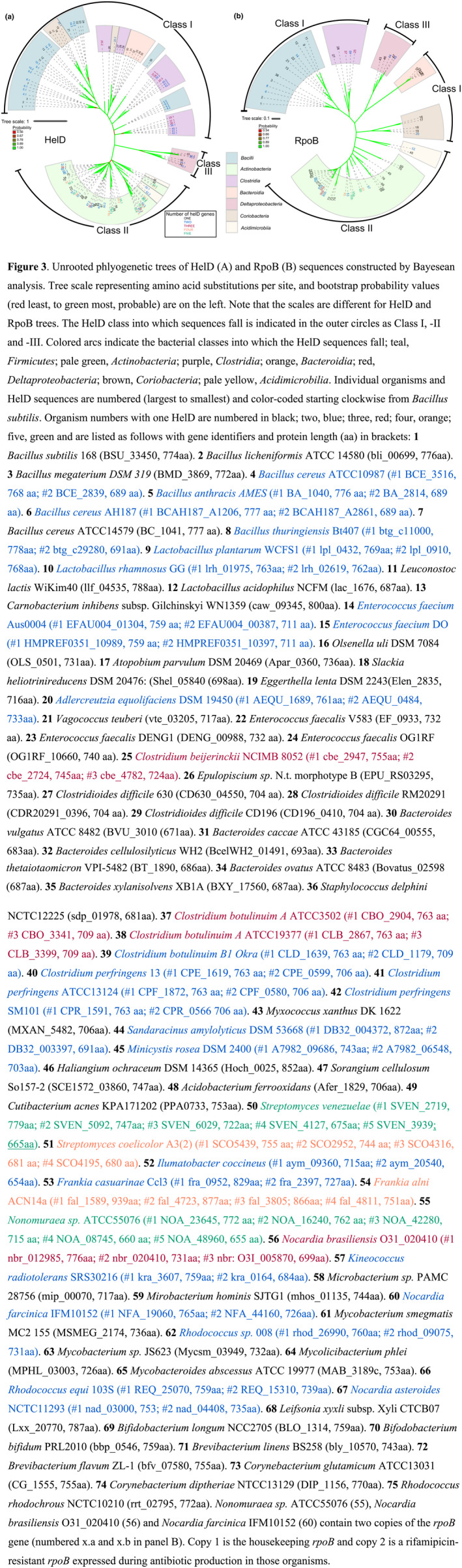
Unrooted phlyogenetic trees of HelD (A) and RpoB (B) sequences constructed by Bayesean analysis. Tree scale representing amino acid substitutions per site, and bootstrap probability values (red least, to green most, probable) are on the left. Note that the scales are different for HelD and RpoB trees. The HelD class into which sequences fall is indicated in the outer circles as Class I, ‐II and ‐III. Colored arcs indicate the bacterial classes into which the HelD sequences fall; teal, *Firmicutes*; pale green, *Actinobacteria*; purple, *Clostridia*; orange, *Bacteroidia*; red, *Deltaproteobacteria*; brown, *Coriobacteria*; pale yellow, *Acidimicrobilia*. Individual organisms and HelD sequences are numbered (largest to smallest) and color‐coded starting clockwise from *Bacillus subtilis*. Organism numbers with one HelD are numbered in black; two, blue; three, red; four, orange; five, green and are listed as follows with gene identifiers and protein length (aa) in brackets: **1** *Bacillus subtilis* 168 (BSU_33450, 774aa). **2** *Bacillus licheniformis* ATCC 14580 (bli_00699, 776aa). **3** *Bacillus megaterium DSM 319* (BMD_3869, 772aa). **4** *Bacillus cereus* ATCC10987 (#1 BCE_3516, 768 aa; #2 BCE_2839, 689 aa). **5** *Bacillus anthracis* AMES (#1 BA_1040, 776 aa; #2 BA_2814, 689 aa). **6** *Bacillus cereus* AH187 (#1 BCAH187_A1206, 777 aa; #2 BCAH187_A2861, 689 aa). **7** *Bacillus cereus* ATCC14579 (BC_1041, 777 aa). **8** *Bacillus thuringiensis* Bt407 (#1 btg_c11000, 778aa; #2 btg_c29280, 691aa). **9** *Lactobacillus plantarum* WCFS1 (#1 lpl_0432, 769aa; #2 lpl_0910, 768aa). **10** *Lactobacillus rhamnosus* GG (#1 lrh_01975, 763aa; #2 lrh_02619, 762aa). **11** *Leuconostoc lactis* WiKim40 (llf_04535, 788aa). **12** *Lactobacillus acidophilus* NCFM (lac_1676, 687aa). **13** *Carnobacterium inhibens* subsp. Gilchinskyi WN1359 (caw_09345, 800aa). **14** *Enterococcus faecium* Aus0004 (#1 EFAU004_01304, 759 aa; #2 EFAU004_00387, 711 aa). **15** *Enterococcus faecium* DO (#1 HMPREF0351_10989, 759 aa; #2 HMPREF0351_10397, 711 aa). **16** *Olsenella uli* DSM 7084 (OLS_0501, 731aa). **17** *Atopobium parvulum* DSM 20469 (Apar_0360, 736aa). **18** *Slackia heliotrinireducens* DSM 20476: (Shel_05840 (698aa). **19** *Eggerthella lenta* DSM 2243(Elen_2835, 716aa). **20** *Adlercreutzia equolifaciens* DSM 19450 (#1 AEQU_1689, 761aa; #2 AEQU_0484, 733aa). **21** *Vagococcus teuberi* (vte_03205, 717aa). **22** *Enterococcus faecalis* V583 (EF_0933, 732 aa). **23** *Enterococcus faecalis* DENG1 (DENG_00988, 732 aa). **24** *Enterococcus faecalis* OG1RF (OG1RF_10660, 740 aa). **25** *Clostridium beijerinckii* NCIMB 8052 (#1 cbe_2947, 755aa; #2 cbe_2724, 745aa; #3 cbe_4782, 724aa). **26** *Epulopiscium sp*. N.t. morphotype B (EPU_RS03295, 735aa). **27** *Clostridioides difficile* 630 (CD630_04550, 704 aa). **28** *Clostridioides difficile* RM20291 (CDR20291_0396, 704 aa). **29** *Clostridioides difficile* CD196 (CD196_0410, 704 aa). **30** *Bacteroides vulgatus* ATCC 8482 (BVU_3010 (671aa). **31** *Bacteroides caccae* ATCC 43185 (CGC64_00555, 683aa). **32** *Bacteroides cellulosilyticus* WH2 (BcelWH2_01491, 693aa). **33** *Bacteroides thetaiotaomicron* VPI‐5482 (BT_1890, 686aa). **34** *Bacteroides ovatus* ATCC 8483 (Bovatus_02598 (687aa). **35** *Bacteroides xylanisolvens* XB1A (BXY_17560, 687aa). **36** *Staphylococcus delphini* NCTC12225 (sdp_01978, 681aa). **37** *Clostridium botulinuim* A ATCC3502 (#1 CBO_2904, 763 aa; #3 CBO_3341, 709 aa). **38** *Clostridium botulinuim* A ATCC19377 (#1 CLB_2867, 763 aa; #3 CLB_3399, 709 aa). **39** *Clostridium botulinuim* B1 Okra (#1 CLD_1639, 763 aa; #2 CLD_1179, 709 aa). **40** *Clostridium perfringens* 13 (#1 CPE_1619, 763 aa; #2 CPE_0599, 706 aa). **41** *Clostridium perfringens* ATCC13124 (#1 CPF_1872, 763 aa; #2 CPF_0580, 706 aa). **42** *Clostridium perfringens* SM101 (#1 CPR_1591, 763 aa; #2 CPR_0566 706 aa). **43** *Myxococcus xanthus* DK 1622 (MXAN_5482, 706aa). **44** *Sandaracinus amylolyticus* DSM 53668 (#1 DB32_004372, 872aa; #2 DB32_003397, 691aa). **45** *Minicystis rosea* DSM 2400 (#1 A7982_09686, 743aa; #2 A7982_06548, 703aa). **46** *Haliangium ochraceum* DSM 14365 (Hoch_0025, 852aa). **47** *Sorangium cellulosum* So157‐2 (SCE1572_03860, 747aa). **48** *Acidobacterium ferrooxidans* (Afer_1829, 706aa). **49** *Cutibacterium acnes* KPA171202 (PPA0733, 753aa). **50** *Streptomyces venezuelae* (#1 SVEN_2719, 779aa; #2 SVEN_5092, 747aa; #3 SVEN_6029, 722aa; #4 SVEN_4127, 675aa; #5 SVEN_3939; 665aa). **51** *Streptomyces coelicolor* A3(2) (#1 SCO5439, 755 aa; #2 SCO2952, 744 aa; #3 SCO4316, 681 aa; #4 SCO4195, 680 aa). **52** *Ilumatobacter coccineus* (#1 aym_09360, 715aa; #2 aym_20540, 654aa). **53** *Frankia casuarinae* Ccl3 (#1 fra_0952, 829aa; #2 fra_2397, 727aa). **54** *Frankia alni* ACN14a (#1 fal_1589, 939aa; #2 fal_4723, 877aa; #3 fal_3805; 866aa; #4 fal_4811, 751aa). **55** *Nonomuraea* sp. ATCC55076 (#1 NOA_23645, 772 aa; #2 NOA_16240, 762 aa; #3 NOA_42280, 715 aa; #4 NOA_08745, 660 aa; #5 NOA_48960, 655 aa). **56** *Nocardia brasiliensis* O31_020410 (#1 nbr_012985, 776aa; #2 nbr_020410, 731aa; #3 nbr: O3I_005870, 699aa). **57** *Kineococcus radiotolerans* SRS30216 (#1 kra_3607, 759aa; #2 kra_0164, 684aa). **58** *Microbacterium sp*. PAMC 28756 (mip_00070, 717aa). **59** *Mirobacterium hominis* SJTG1 (mhos_01135, 744aa). **60** *Nocardia farcinica* IFM10152 (#1 NFA_19060, 765aa; #2 NFA_44160, 726aa). **61** *Mycobacterium smegmatis* MC2 155 (MSMEG_2174, 736aa). **62** *Rhodococcus* sp. 008 (#1 rhod_26990, 760aa; #2 rhod_09075, 731aa). **63** *Mycobacterium sp*. JS623 (Mycsm_03949, 732aa). **64** *Mycolicibacterium phlei* (MPHL_03003, 726aa). **65** *Mycobacteroides abscessus* ATCC 19977 (MAB_3189c, 753aa). **66** *Rhodococcus equi* 103S (#1 REQ_25070, 759aa; #2 REQ_15310, 739aa). **67** *Nocardia asteroides* NCTC11293 (#1 nad_03000, 753; #2 nad_04408, 735aa). **68** *Leifsonia xyxli* subsp. Xyli CTCB07 (Lxx_20770, 787aa). **69** *Bifidobacterium longum* NCC2705 (BLO_1314, 759aa). **70** *Bifodobacterium bifidum* PRL2010 (bbp_0546, 759aa). **71** *Brevibacterium linens* BS258 (bly_10570, 743aa). **72** *Brevibacterium flavum* ZL‐1 (bfv_07580, 755aa). **73** *Corynebacterium glutamicum* ATCC13031 (CG_1555, 755aa). **74** *Corynebacterium diptheriae* NTCC13129 (DIP_1156, 770aa). **75** *Rhodococcus rhodochrous* NCTC10210 (rrt_02795, 772aa). *Nonomuraea sp.* ATCC55076 (55), *Nocardia brasiliensis* O31_020410 (56) and *Nocardia farcinica* IFM10152 (60) contain two copies of the *rpoB* gene (numbered x.a and x.b in panel B). Copy 1 is the housekeeping *rpoB* and copy 2 is a rifamipicin‐resistant *rpoB* expressed during antibiotic production in those organisms.

Searching for HelD‐like sequences using the conserved domain architecture retrieval tool (CDART; NCBI) portal identified >13,000 hits. Additional searches using NCBI BLASTP suggest that there are substantially more sequences in the database, but many of these are from incomplete genomes and/or metagenomic sequencing projects, making systematic identification and classification of sequences unfeasible, particularly in cases where an organism carries more than one *helD* gene (see below). Nevertheless, it is clear that HelD is widely distributed in the eubacteria, especially in the *Firmicutes* and *Actinobacteria* phyla of the Gram‐positive eubacterial domain. To date, we have not detected HelD‐like sequences in *Archaea* or *Eucarya*. Previously, Newing et al. (Newing et al., 2020) showed that HelD sequences fall into two classes, which was confirmed at the structural and functional level in comparing HelD proteins from the *Firmicutes* and *Actinobacteria* (Kouba et al., 2020; Newing et al., 2020; Pei et al., 2020). Using a wider range of carefully curated sequences from complete genomes identified from the initial CDART search, an unrooted phylogenetic tree was constructed to enable a more detailed understanding of HelD distribution and phylogeny which was compared against the RNAP RpoB (β) subunit (Figure 3; note different tree scales).

Four features are clear from this tree (Figure 3a): 1. HelD is also present in Gram‐negative bacteria; 2. The third class of HelD is present in the *Deltaproteobacteria*; 3. In some organisms HelD has been ancestrally acquired by horizontal gene transfer; 4. Many organisms contain more than one *helD* gene, with the *Firmicutes*, *Clostridia*, *Acidimicrobiia*, and *Deltaproteobacteria* having up to three, and the *Actinobacteria* up to five.

**FIGURE A1:**
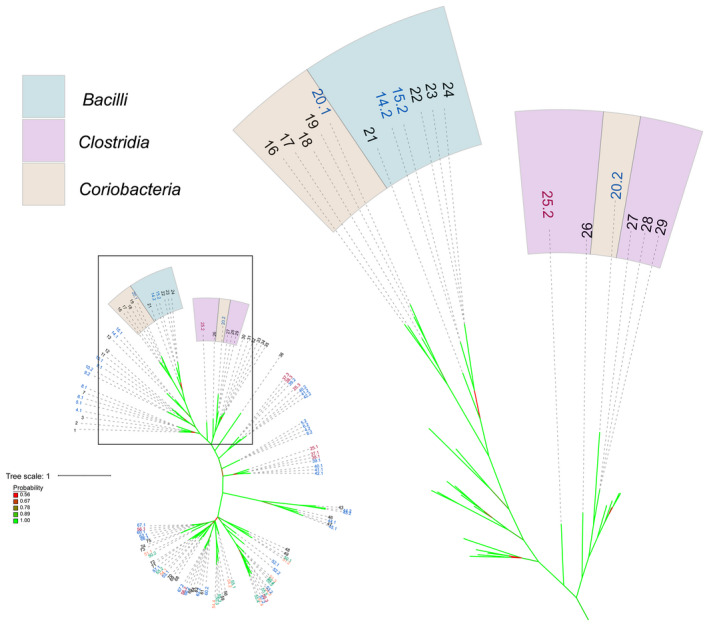
Acquisition of *helD* genes by *Coriobacteria* from *Firmicutes* and *Clostridia*. The phylogenetic tree from Figure 1 is shown on the left side with the region boxed expanded on the right side. Bacterial classes are colored, species numbered, number of *helD* genes colored as in Figure 1: *Bacilli*, teal; *Clostridia*, purple; *Coriobacteria*, brown. **14** *Enterococcus faecium* Aus0004 (#2 EFAU004_00387, 711 aa). **15** *Enterococcus faecium* DO (#2 HMPREF0351_10397, 711 aa). **16** *Olsenella uli* DSM 7084 (OLS_0501, 731aa). **17** *Atopobium parvulum* DSM 20469 (Apar_0360, 736aa). **18** *Slackia heliotrinireducens* DSM 20476: (Shel_05840 (698aa). **19** *Eggerthella lenta* DSM 2243(Elen_2835, 716aa). **20** *Adlercreutzia equolifaciens* DSM 19450 (#1 AEQU_1689, 761aa; #2 AEQU_0484, 733aa). **21** *Vagococcus teuberi* (vte_03205, 717aa). **22** *Enterococcus faecalis* V583 (EF_0933, 732 aa). **23** *Enterococcus faecalis* DENG1 (DENG_00988, 732 aa). **24** *Enterococcus faecalis* OG1RF (OG1RF_10660, 740 aa). **25** *Clostridium beijerinckii* NCIMB 8052 (#2 cbe_2724, 745aa). **26** *Epulopiscium sp*. N.t. morphotype B (EPU_RS03295, 735aa). **27** *Clostridioides difficile* 630 (CD630_04550, 704 aa). **28** *Clostridioides difficile* RM20291 (CDR20291_0396, 704 aa). **29** *Clostridioides difficile* CD196 (CD196_0410, 704 aa). One *helD* gene, black; two, blue; three, red

**FIGURE A2:**
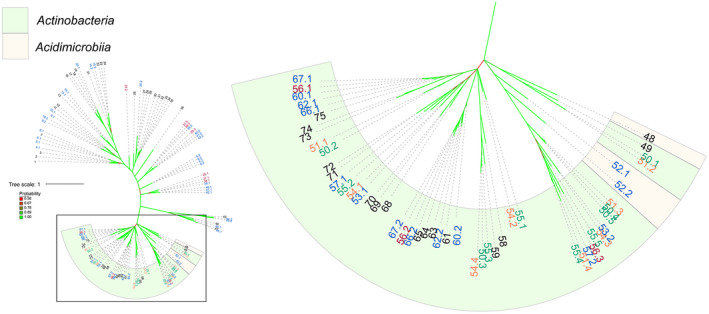
Acquisition of *helD* genes by *Acidimicrobiia* from *Actinobacteria*. The phylogenetic tree from Figure 1 is shown on the left side with the region boxed expanded on the right side. Bacterial classes are colored, species numbered, number of *helD* genes colored as in Figure 1: *Actinobacteria*, pale green; *Acidimicrobiia*, pale yellow. **48** *Acidobacterium ferrooxidans* (Afer_1829, 706aa). **49** *Cutibacterium acnes* KPA171202 (PPA0733, 753aa). **50** *Streptomyces venezuelae* (#1 SVEN_2719, 779aa; #2 SVEN_5092, 747aa; #3 SVEN_6029, 722aa; #4 SVEN_4127, 675aa; #5 SVEN_3939; 665aa). **51** *Streptomyces coelicolor* A3(2) (#1 SCO5439, 755 aa; #2 SCO2952, 744 aa; #3 SCO4316, 681 aa; #4 SCO4195, 680 aa). **52** *Ilumatobacter coccineus* (#1 aym_09360, 715aa; #2 aym_20540, 654aa). **53** *Frankia casuarinae* Ccl3 (#1 fra_0952, 829aa; #2 fra_2397, 727aa). **54** *Frankia alni* ACN14a (#1 fal_1589, 939aa; #2 fal_4723, 877aa; #3 fal_3805; 866aa; #4 fal_4811, 751aa). **55** *Nonomuraea* sp. ATCC55076 (#1 NOA_23645, 772 aa; #2 NOA_16240, 762 aa; #3 NOA_42280, 715 aa; #4 NOA_08745, 660 aa; #5 NOA_48960, 655 aa). **56** *Nocardia brasiliensis* O31_020410 (#1 nbr_012985, 776aa; #2 nbr_020410, 731aa; #3 nbr: O3I_005870, 699aa). **57** *Kineococcus radiotolerans* SRS30216 (#1 kra_3607, 759aa; #2 kra_0164, 684aa). **58** *Microbacterium sp*. PAMC 28756 (mip_00070, 717aa). **59** *Mirobacterium hominis* SJTG1 (mhos_01135, 744aa). **60** *Nocardia farcinica* IFM10152 (#1 NFA_19060, 765aa; #2 NFA_44160, 726aa). **61** *Mycobacterium smegmatis* MC2 155 (MSMEG_2174, 736aa). **62** *Rhodococcus* sp. 008 (#1 rhod_26990, 760aa; #2 rhod_09075, 731aa). **63** *Mycobacterium sp*. JS623 (Mycsm_03949, 732aa). **64** *Mycolicibacterium phlei* (MPHL_03003, 726aa). **65** *Mycobacteroides abscessus* ATCC 19977 (MAB_3189c, 753aa). **66** *Rhodococcus equi* 103S (#1 REQ_25070, 759aa; #2 REQ_15310, 739aa). **67** *Nocardia asteroides* NCTC11293 (#1 nad_03000, 753; #2 nad_04408, 735aa). **68** *Leifsonia xyxli* subsp. Xyli CTCB07 (Lxx_20770, 787aa). **69** *Bifidobacterium longum* NCC2705 (BLO_1314, 759aa). **70** *Bifodobacterium bifidum* PRL2010 (bbp_0546, 759aa). **71** *Brevibacterium linens* BS258 (bly_10570, 743aa). **72** *Brevibacterium flavum* ZL‐1 (bfv_07580, 755aa). **73** *Corynebacterium glutamicum* ATCC13031 (CG_1555, 755aa). **74** *Corynebacterium diptheriae* NTCC13129 (DIP_1156, 770aa). **75** *Rhodococcus rhodochrous* NCTC10210 (rrt_02795, 772aa). One *helD* gene, black; two, blue; three, red; four, orange; five, green

Overall, the tree contains three major branches: Class I HelD sequences originating mainly from the low G+C Gram‐positives and *Bacteriodia*, Class II HelD sequences from the high G+C Gram‐positives, and a novel Class III identified in *Deltaproteobacteria*. Interestingly, the HelD sequences from the Actinobacterial *Coriobacteria* class, typified by *Olsenella uli* that is associated with gingivitis, are all located to the Class I branch of the tree (numbers 16–20; Figure 3). Branch divergence and clustering of sequences to regions of the tree comprising *Lactobacilli* (numbers 14, 15, 21–24; Figure 3) and *Clostridia* (numbers 25–29; Figure 3) indicate that an ancestral *Coriobacteria* likely acquired *helD* genes by horizontal gene transfer from these organisms (Appendix 1; Figure A1). That *Coriobacteria* is isolated from the gingival crevice, gastrointestinal and genital tracts (Clavel et al., 2014) is consistent with this proposition. The length of the branches suggests this horizontal transfer event occurred long ago but after the evolution of the mammalian hosts that provide environments with co‐localised *Lactobacilli*, and that *helD* genes have been stably inherited and co‐evolved within the *Coriobacteria*. In addition to the *helD* gene from *Adlercreutzia equolifaciens* DSM 19450 (AEQU_1689, number 20.1; Figure 3) that clusters with those of the other *Coriobacteria*, *A*. *equolifaciens* contains a second *helD* gene (AEQU_0484, number 20.2; Figure 3) that clusters with *Clostridia*, suggesting it may have been acquired through a separate horizontal gene transfer event rather than through duplication and evolution of a gene inherited by a single acquisition event (Appendix 1; Figure A1). The fact that *Lactobacilli*, *Clostridia*, and *Aldercreutzia* all inhabit the gastrointestinal tract makes this a reasonable hypothesis. There is also some evidence that Class II HelD sequences have been acquired by horizontal gene transfer between the *Actinobacteria* to the *Acidimicrobiia* (numbers 48, 52.1, and 52.2; Figure 3 and Appendix 1 Figure A2). The *Acidimicrobiia* is a recently described class, exemplified by *Acidobacterium ferrooxidans* (number 48; Figure 3) that have been isolated from diverse, but generally acidic and hostile environments, and tend to grow slowly which may account for the paucity of information and diversity of species currently available. At least one species of the *Acidimicrobiia*, *Ilumatobacter coccineus* (number 52, Figure 3) contains multiple copies of *helD*.

Comparison of the phylogenetic tree of the RNA polymerase β subunit RpoB with the HelD tree supports this assumption that *helD* genes in the *Coriobacteria* and *Acidimicrobiia* have been acquired by horizontal gene transfer from *Firmicutes*/*Clostridia*/*Actinobacteria* that share the same ecological niches (Figure 3a and b). Acquisition of *helD* genes by horizontal gene transfer in the *Bacteroidia* is described below.

### 3.2 Acquisition of *helD* in Gram‐negative *Bacteroides*

**FIGURE A3:**
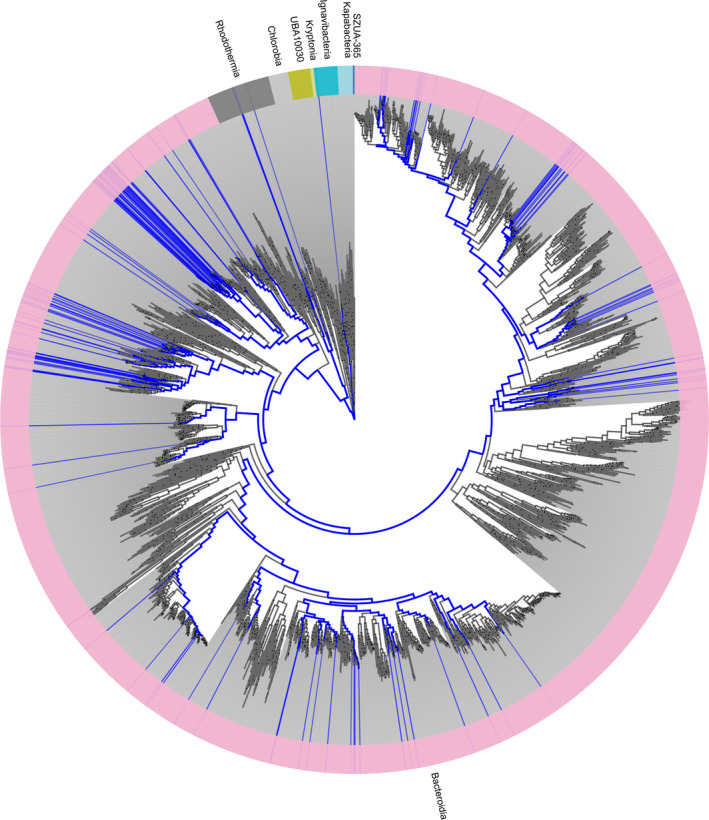
Distribution of helD genes in the phylum Bacteroidota. HelD sequences from Bacteroidota RefSeq genomes were retrieved from a BLASTP search and mapped to individual species within the phylum Bacteroidota using Annotree (Mendler et al.,^2019^). Bacteroidotal classes are shown in the colored outer ring with Bacteroidia in pink, Rhodothermia in grey, Chlorobia in light grey, UBA10030 in lime green, Kryptonia in pale green, Ignavibacteria in cyan, Kapabacteria in pale blue, and SZUA‐365 in blue. Individual species are shown as lines radiating out from the circular dendrogram with species containing HelD sequences highlighted in bright blue

**FIGURE A4:**
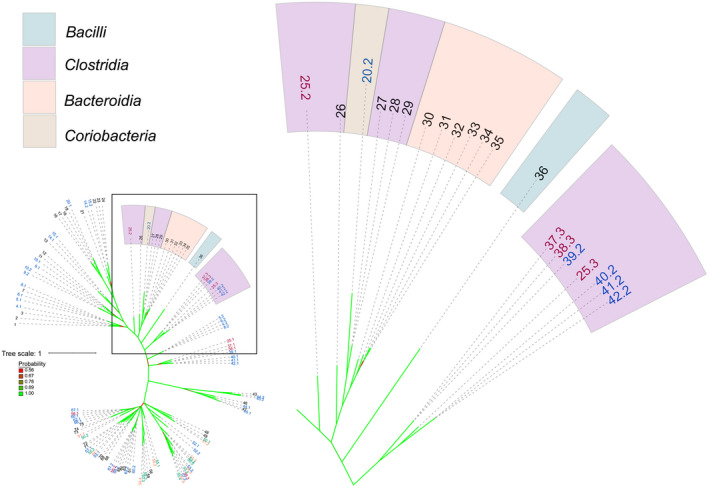
Acquisition of *helD* genes by *Bacteroides* from *Clostridia*. The phylogenetic tree from Figure 1 is shown on the left side with the region boxed expanded on the right side. Bacterial classes are colored, species numbered, number of *helD* genes colored as in Figure 1: *Bacilli*, teal; *Clostridia*, purple; *Bacteroides*, orange; *Coriobacteria*, brown. **20** *Adlercreutzia equolifaciens* DSM 19450 (#2 AEQU_0484, 733aa). **25** *Clostridium beijerinckii* NCIMB 8052 (#2 cbe_2724, 745aa; #3 cbe_4782, 724aa). **26** *Epulopiscium sp*. N.t. morphotype B (EPU_RS03295, 735aa). **27** *Clostridioides difficile* 630 (CD630_04550, 704 aa). **28** *Clostridioides difficile* RM20291 (CDR20291_0396, 704 aa). **29** *Clostridioides difficile* CD196 (CD196_0410, 704 aa). **30** *Bacteroides vulgatus* ATCC 8482 (BVU_3010 (671aa). **31** *Bacteroides caccae* ATCC 43185 (CGC64_00555, 683aa). **32** *Bacteroides cellulosilyticus* WH2 (BcelWH2_01491, 693aa). **33** *Bacteroides thetaiotaomicron* VPI‐5482 (BT_1890, 686aa). **34** *Bacteroides ovatus* ATCC 8483 (Bovatus_02598 (687aa). **35** *Bacteroides xylanisolvens* XB1A (BXY_17560, 687aa). **36** *Staphylococcus delphini* NCTC12225 (sdp_01978, 681aa). **37** *Clostridium botulinuim* A ATCC3502 (#3 CBO_3341, 709 aa). **38** *Clostridium botulinuim* A ATCC19377 (#3 CLB_3399, 709 aa). **39** *Clostridium botulinuim* B1 Okra (#2 CLD_1179, 709 aa). **40** *Clostridium perfringens* 13 (#2 CPE_0599, 706 aa). **41** *Clostridium perfringens* ATCC13124 (#2 CPF_0580, 706 aa). **42** *Clostridium perfringens* SM101 (#2 CPR_0566 706 aa). One *helD* gene, black; two, blue; three, red

**FIGURE A5:**
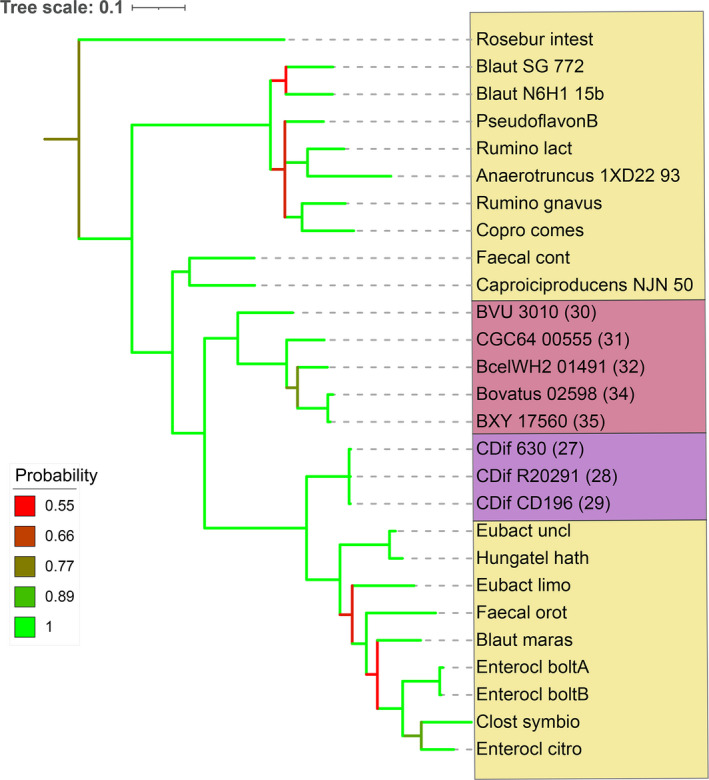
Phylogenetic tree of HelD sequences from Bacteroides and Clostridia. Tree scale and bootstrap values are shown at the top and left, respectively. Colored boxes denote cluster IV and XIVa Clostridia (yellow), cluster IX Clostridia (purple), and Bacteroides (red). The numbers in parentheses correspond to the organisms used in Figure 1. Roseburia intestinalis (Rosebur intest), *Blautia* sp. SG‐772 (Blaut SG772), *Blautia* sp. N6H1‐15 (Blaut N6H1‐15b), *Pseudoflavonifractor* sp. BSD2780061688st1 E11 (PseudoflavonB), *Ruminococcus lactaris* (Rumino lact), *Anaerotruncus* sp. 1XD22‐93 (Anaerotruncus 1XD22‐93), *Ruminococcus gnavus* (*Rumino gnavus*), *Coprococcus comes* (Copro comes), *B. vulgatus* ATCC 8482 (BVU 3010), B. caccae ATCC 43185 (CGC64 00555), *B. cellulosilyticus* WH2 (BcelWH2 01491), *B. ovatus* ATCC 8483 (Bovatus 02598), *B. xylanisolvens* XB1A (BXY 17560), *C. difficile* 630 (CDif 630), *C. difficile* RM20291 (CDif R20291), *C. difficile* CD196 (CDif CD196), *Faecalicatena contorta* (Faecal cont), *Caproiciproducens* sp. NJN‐50 (Caproiciproducens NJN‐50), *Eubacterium uniforme* (Eubact uncl), *Hungatella hathewayi* (*Hungatel hath*), *Eubacterium limosum* (Eubact limo), *Faecalicatena orotica* (Faecal orot), *Blautia marasmi* (Blaut maras), *Enterocloster bolteae* (Enterocl boltA and B), *Clostridium symbiosum* (Clost symbio), and *Enterocloster citroniae* (Enterocl citro)

HelD sequences were also identified in the phylum of Gram‐negative bacteria, *Bacteroides*. Mapping sequences to the phylum *Bacteroidota* shows HelD is widely distributed throughout the class *Bacteroidia* with additional representation in the classes *Rhodothermia* and *Ignavibacteria* (Appendix 1; Figure A3). Phylogenetically, many of *the Bacteroidial* HelD sequences clustered close to HelD sequences from *Clostridioides difficile* (Figure 3a and Appendix 1 Figure A4; sequences 27–29 *C*. *difficile*, 30–35 *Bacteroides*). Extended analysis indicated that HelD sequences from *Bacteroides* and *Parabacteroides* (family *Porphyromonadaceae*) clustered closest to those from *Firmicutes* that are strict gut anaerobes from the order *Clostridiales* (Appendix 1; Figure A5). These bacteria were from cluster IV (*Ruminoccoaceae*) and XIVa (*Lachnospiraceae*) that are abundant gut microbes associated with many aspects of good health, and the cluster XI gut pathogen *C*. *difficile* (Lopetuso et al., 2013; Lozupone et al., 2012; Milani et al., 2017). Since the *Bacteroides* and *Parabacteroides* are also abundant obligate gut anaerobes, this clustering suggested that *helD* was horizontally transferred from an anaerobic gut *Firmicute*, most likely from the order *Clostridiales* (Appendix 1; Figure A5). Analysis of the genome context of *helD* genes indicated they were not (or are no longer) located in mobile genetic elements, except for *B*. *thetaiaotamicron*, and along with their widespread distribution in *Bacteroides*/*Parabacteroides* suggests *helD* genes have been retained over a significant period, indicating they serve a useful cellular function. The fact that HelD sequences identified in *Bacteroides* cluster with Class I sequences from the low G+C Gram‐positive bacteria rather than forming a separate Class, as seen with HelD from the *Deltaproteobacteria* (see below), further supports the idea that this group acquired *helD* genes by horizontal gene transfer due to sharing a similar environmental niche to anaerobic gut *Clostridiales*.

### 3.3 A novel HelD class in Gram‐negative bacteria

**FIGURE 4:**
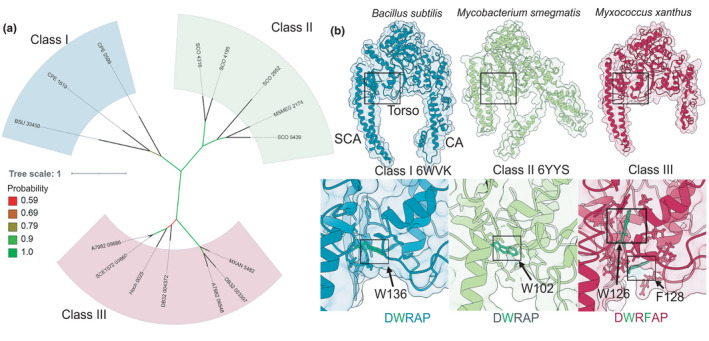
Three classes of HelD. Panel A shows a focused unrooted phylogenetic tree constructed using HelD sequences, with numbers (#) as used in Figure 1A: *B*. *subtilis* 168, BSU [#1]; *C*. *perfringens* 13, CPE [#40]; *S*. *coelicolor* A3(2), SCO [#51]; *M*. *smegmatis* MC2 155, MSMEG [#61], and *Deltaproteobacterial* sequences from *M*.* xanthus* DK 1622, MXAN [#43]; *S*. *amylolyticus* DSM 53668, DB32 [#44]; *M*. *rosea* DSM 2400, A7982 [#45]; *H*. *ochraceum* DSM 14365, Hoch [#46]; *S*. *cellulosum* So157‐2, SCE1572 [#47]. Tree scale representing amino acid substitutions per site, and bootstrap values are shown on the left. The coloring of bacterial classes is the same as in Figure 1. Panel B shows structures (ribbons and transparent surface representations) of whole HelD (top) and Trp‐cage regions (bottom) of Class I (*B*. *subtilis* PDB ID 6WVK), Class II (*M*. *smegmatis* PDB ID 6YYS), and Class III (*M*. *xanthus*, homology model) using the same color scheme for bacterial classes as in Figures 1 and 2A. Conserved Trp (all classes) and additional amino acid (Class III) are shown as green sticks, with other amino acids that form the cage shown in the appropriate color for their class

**FIGURE A6:**
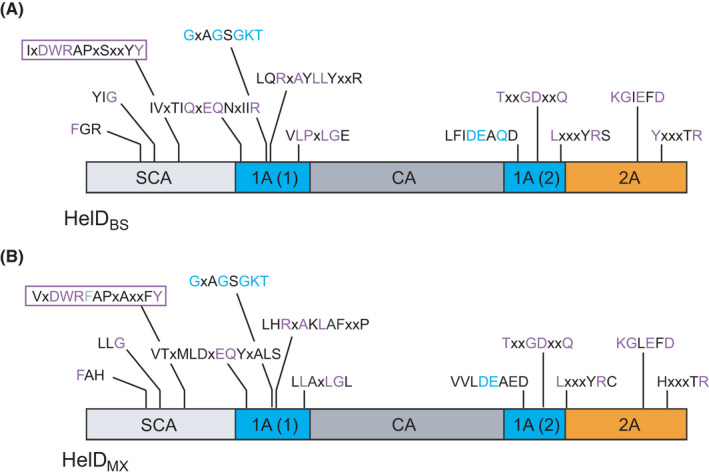
Conserved HelD sequence motifs. Panel A shows a schematic of B. subtilis HelD domain organization with conserved sequence motifs adapted from Newing et al., (Newing et al., 2020), with panel B showing the equivalent sequence motifs from *M. xanthus* HelD. Appendix Table A1 shows the conserved sequence motifs with sequence numbers referring to the B. subtilis HelD sequence. X corresponds to a poorly conserved sequence (any amino acid) and h to a conserved hydrophobic residue. Residues colored red are specific to class I and green to class II sequences. The HelD motifs from the Class III *M. xanthus* HelD (Class IIIMX) are shown in the right column with absolutely conserved motif residues shown in purple (blue for the ATP binding motifs) and the Class III defining residue (F in the case of M. xanthus) that is inserted in the DWRAP motif shown in grey (see text for more details)

The analysis presented in this work also shows that there is a third class of HelD proteins encoded by the *Deltaproteobacteria* (Class III, Figures 3 and 4; see below). Newing et al. (Newing et al., 2020) identified Class I and II HelD proteins based on the conservation of twelve sequence motifs. These motifs (labeled I‐XII, Appendix 1; Figure A6) are all conserved in Class III proteins (exemplified by *Myxococcus xanthus* HelD), despite the low overall levels of sequence similarity found in HelD proteins (Newing et al., 2020). A model of *M*. *xanthus* HelD was also generated from an unbiased screen of the protein structure database (Figure 4; see Materials and Methods). As seen with Class I and II proteins, there is a HelD‐specific N‐terminal domain of ~50–150 amino acids that has a long antiparallel α‐helical structure (secondary channel arm, SCA, Figure 4b) that is required to anchor HelD in the secondary channel of its cognate RNAP (Kouba et al., 2020; Newing et al., 2020; Pei et al., 2020), and the 1A helicase domain is split by the insertion of an arm‐like structure (clamp arm, CA, Figure 4b and S6) that is used to bind within the primary channel of RNAP, forcing it open to aid the release of bound nucleic acids (Kouba et al., 2020; Newing et al., 2020; Pei et al., 2020).

**FIGURE 5:**
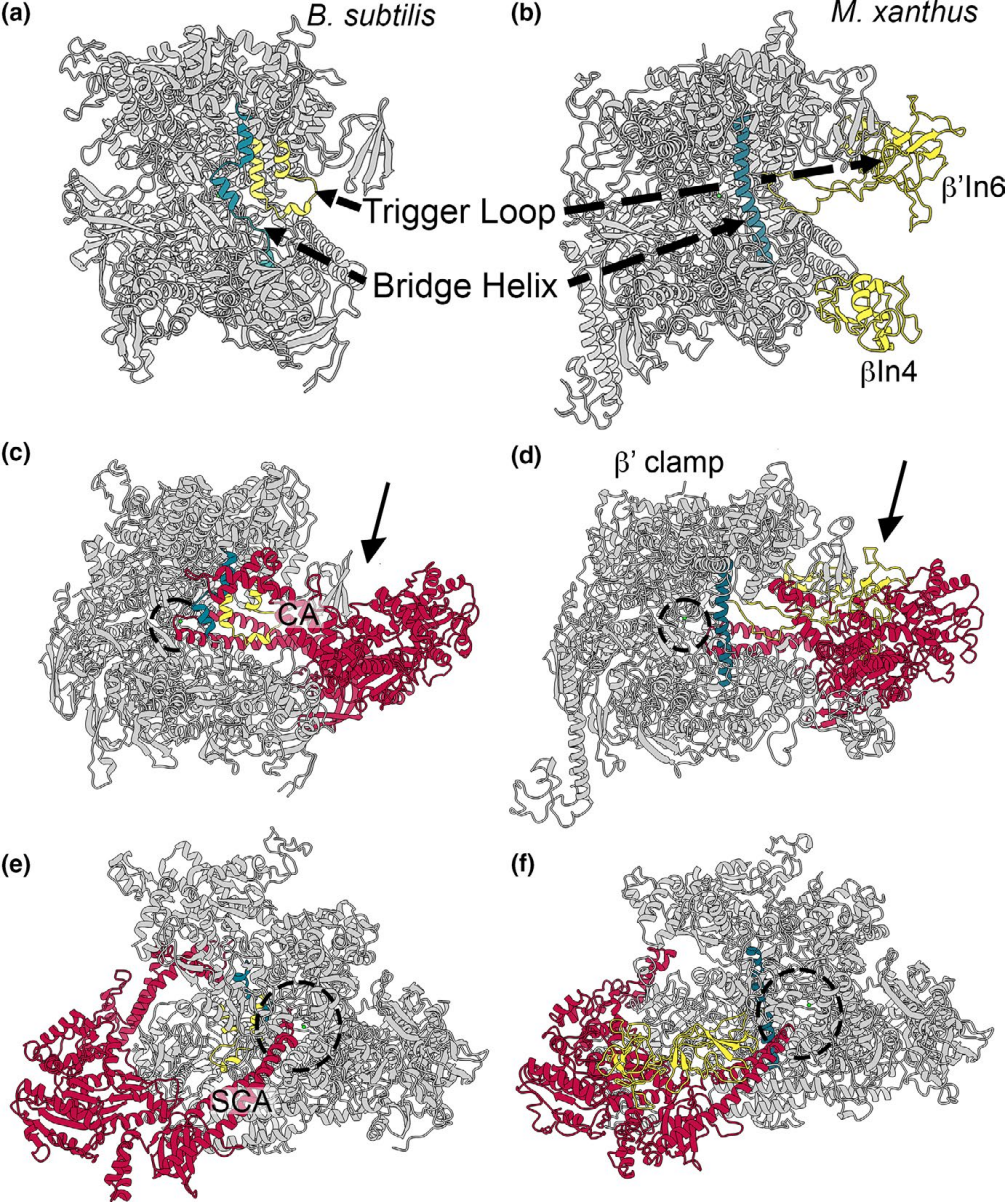
Comparison of *B*. *subtilis* RNAP–HelD complex with the *M*.* xanthus* model. Panels A and B show structures of *B*. *subtilis* (PDB ID 6WVK) and *M*.* xanthus* (model) RNAPs in complex with HelD, respectively, in which HelD has been removed to more clearly visualize elements referred to in the text. The trigger loop (yellow) and bridge helix (teal) are indicated along with the lineage‐specific βIn4 (also yellow) and β’In6 inserts in the *M*.* xanthus* model. Panels C and E show the *B*. *subtilis* RNAP–HelD complex. Panels D and F show *M*.* xanthus* RNAP–HelD model. RNAP is shown in grey in all panels, HelD in red, bridge helix in teal, and trigger loop in yellow (see text for further details). The active site Mg^2+^ is shown as a small green sphere (within the dotted circles). The arrows in panels C and E denote the view of the respective RNAP–HelD complex in panels E and F. The view in panels C and D is into the primary channel to which the clamp arm (CA) of HelD binds. The view in panels E and F is into the secondary channel (dotted circle) into which the secondary channel arm (SCA) is inserted

An absolutely conserved DWR (Asp‐Trp‐Arg) sequence motif was identified in the unique N‐terminal domain of all HelD sequences, and determination of the structures of HelD showed that the conserved Trp residue resides within a hydrophobic pocket called the Trp‐cage, important in stabilizing the interaction between the N‐terminal domain wedged deep into the secondary channel of RNAP and the helicase 1A domain (Newing et al., 2020). In most HelD sequences identified to date, the DWR motif is extended to DWR[A/S]P, but in *Deltaproteobacterial* HelDs there is an additional amino acid inserted in this motif following the R residue, i.e., DWRX[A/S]P, which is a key defining feature of a Class III HelD (Appendix 1; Figure A6). This additional amino acid does not appear to be highly conserved, the motif being DWRFAP in *M*. *xanthus*, DWRNAP in *Haliangum ocraceum*, and DWRHAP in *Sorangium cellulosum*, with H or N appearing to be most common. Modeling suggests this amino acid is located on a loop with its side chain in an additional pocket that may be important in reinforcing the connection between the SCA and torso, potentially through burying the conserved Trp deeper inside the Trp‐cage in comparison with Class I and II HelDs (boxed green residues, Figure 4b). Structural modeling also shows the SCA of *M*.* xanthus* HelD (HelD_MX_) is longer than that of *M*. *smegmatis* (HelD_MS_) but shorter than the *B*. *subtilis* protein (HelD_BS_). The tip of the SCA of HelD_MX_ does not reach the active site (catalytic Mg^2+^, green sphere; compare dashed circles in Figure 5c–f) but would clash with the bridge helix in RNAP (teal, Figure 5d and f), potentially causing it to distort and displace the template DNA strand as seen with HelD_BS_ (Newing et al., 2020). The RNAP trigger loop contains a large insertion in the *Deltaproteobacteria* (β’In6, Figure 5b) similar to that seen in *Gammaproteobacteria*, and it was assumed this (and the βIn4 insertion, Figure 5b) would sterically interfere with HelD binding to RNAP in Gram‐negative bacteria. Although the trigger loop in the modeled *M*.* xanthus* RNAP–HelD complex does clash with HelD_MX_ (Figure 5e and f), this is not extensive, and given the inherent flexibility in this domain, small conformational changes would readily enable binding as seen in Gram‐positive bacteria (Kouba et al., 2020; Newing et al., 2020; Pei et al., 2020). The CA of HelD_MX_ is similar in size to that of HelD_MS_ (although it does not contain a PCh domain; Figure 4b). The CA domain is required for clamp opening and DNA release in the Gram‐positive systems, and likely will serve a similar function in Class III HelDs.

Examination of sequences retrieved from the CDART search indicated *helD* genes may be even more widely distributed in the *Proteobacteria* (including the *Gammaproteobacteria*), although this could not be verified by searches of complete genomes in databases such as KEGG and may represent misclassification from metagenomic sequencing projects. For example, BLASTP searches suggest hits reported as being from *E*. *coli* and *Vibrio vulnificus* identified from metagenomic data are in fact from *Bacteroides* and *Bacillus*, respectively (Poyet et al., 2019), and NCBI SRA accession code: PRJNA523266). Nevertheless, helD genes may be more widely distributed in *Proteobacteria*.

### 3.4 RNAP δ subunit and HelD

**FIGURE 6:**
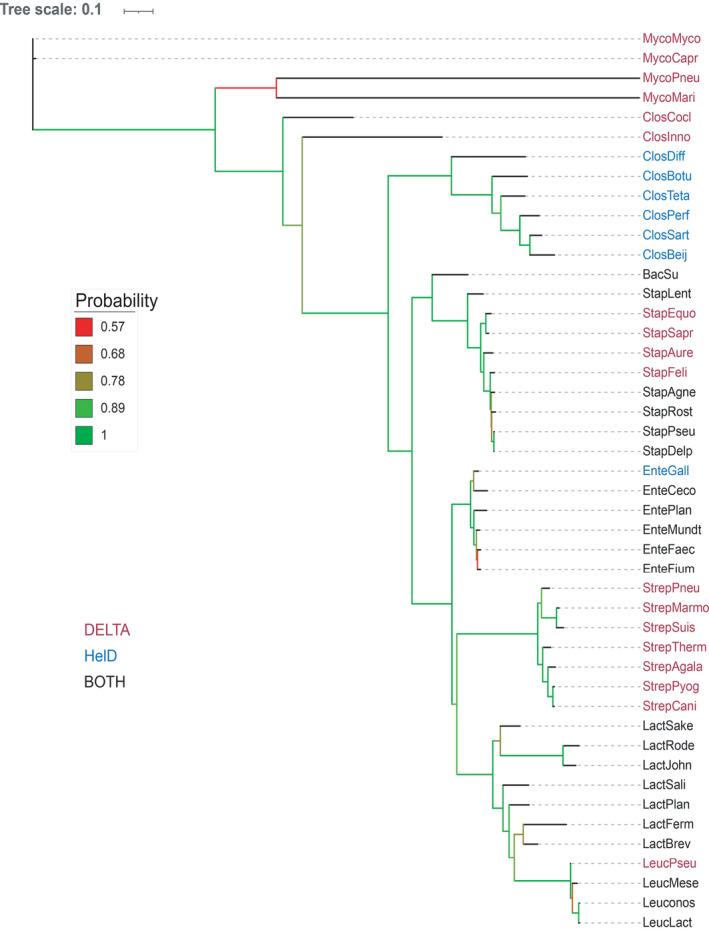
Phylogenetic tree of RpoB with respect to the distribution of HelD and the d subunit of RNAP. Tree scale and bootstrap values are shown on the left. Organisms that contain the d subunit (DELTA) are shown in red, just HelD (blue) and both d and HelD (black). *Mycoplasma mycoides* (MycoMyco), *Mycoplasma capricolum* (MycoCapr), *Mycoplasma pneumoniae* (MycoPneu), *Mycoplasma marinum* (MycoMari), *Erysipelatoclostridium cocleatum* (ClosCocl), *Erysipelatoclostridium inoccuum* (ClosInno), *Clostridioides difficile* (ClosDiff), *Clostridium botulinum* (ClosBotu), *Clostridium perfringens* (ClosPerf), *Clostridium sartagoforme* (ClosSart), *Clostridium beijernickii* (ClosBeij), *Bacillus subtilis* (BacSu), *Staphylococcus lentus* (StapLent), *Staphylococcus equorum* (StaphEquo) *Staphylococcus saprophyticus* (StapSapr), *Staphylococcus aureus* (StapAure), *Staphylococcus felis* (StapFeli), *Staphylococcus agnetis* (StapAgne), *Staphylococcus rostri* (Staprost), *Staphylococcus pseudointermidius* (StapPseu), *Staphylococcus delphini* (StapDelp), *Enterococcus gallinarum* (EnteGall), *Enterococcus cecorum* (EnteCeco), *Enterococcus plantarum* (EntePlan), *Enterococcus mundti* (EnteMundt), *Enterococcus faecalis* (EnteFaec), *Enterococcus faecium* (EnteFium), *Streptococcus pneumoniae* (StrepPneu), *Streptococcus marmotae* (StrepMarmo), *Streptococcus suis* (StrepSuis), *Streptococcus thermophilus* (StrepTherm), *Streptococcus agalactiae* (StrepAgala), *Streptococcus pyogenes* (StrepPyog), *Streptococcus canis* (StrepCani), *Lactobacillus sakei* (LactSake), *Lactococcus rodentium* (LactRode), *Lactobacillus johnsonii* (LactJohn), *Lactobacillus salivarius* (LactSali), *Lactobacillus plantarum* (LactPlan), *Lactobacillus fermentum* (LactFerm), *Lactobacillus brevis* (LactBrev), *Leuconostoc pseudomesenteroides* (LeucPseu), *Leuconostoc mesenteroides* (LeucMese), *Leuconostoc sp*. (Leuconos), and *Leuconostoc lactis* (LeucLact)

The *Firmicutes* have the smallest multi‐subunit RNAPs currently known (Lane & Darst, 2010a, 2010b), as well as auxiliary subunits δ and ε that are not found in other bacteria (Keller et al., 2014; Weiss & Shaw, 2015). In the original work characterizing the function of HelD as a transcription complex recycling factor, it was shown that although δ or HelD on their own enhanced recycling, there was a synergistic relationship between them in *B*. *subtilis* transcription recycling assays (Wiedermannova et al., 2014). Structural analysis of RNAP recycling complexes shows that δ and HelD interact, as well as providing clues as to how δ could enhance the recycling activity of HelD by augmenting clamp opening (Pei et al., 2020). These structural studies also provided insights into how δ could facilitate transcription recycling in the absence of HelD (Miller et al., 2021). Genome searches indicated that not all *Firmicutes* contained both *helD* and *rpoE* (encoding the δ subunit) genes, and an analysis was performed based on the *rpoB* gene to establish whether there is segregation of genes amongst orders and/or based on the natural environment (Figure 6).

In the bulk of cases, the *Bacilli*, *Lactobacilli*, *Leuconostoc*, and *Enterococci* contained genes for both HelD and δ, and if the gene for one protein was missing, the other was present (Figure 6). The *Staphylococci* were heterogeneous with species such as *S*. *rostri* containing both *helD* and *rpoE* genes, whereas *S*. *aureus* only contained the gene for the δ subunit. There is a segregation of species containing both *helD* and *rpoE cf*. *rpoE* only, with *rpoE* only present in the *S*. *saprophyticus* and *S*. *aureus* clusters (Takahashi et al., 1999). Species that fall within the *S*.* hyicus*‐*intermedius* cluster (*e*.*g*., *S*. *rostri*) contained both *helD* and *rpoE*, but there were exceptions such as *S*. *felis*, which only contained *rpoE* (Figure 6). The *Streptococci* (order *Lactobacillales*) only contained the *rpoE* gene (Figure 6), whereas the *Clostridia*, except for *C*. (*Erysipelatoclostridium*) *cocleatum* and *inoccuum*, only contained *helD* genes (Figure 6). Thus, it appears that in the *Firmicutes*, especially class *Bacillus*, the default situation is for both *rpoE* and *helD* to be present, but the absence of one gene is compensated for by the presence of the other to ensure the ability to recycle stalled transcription complexes is retained.

### 3.5 Many bacteria contain multiple *helD* genes

**FIGURE A7:**
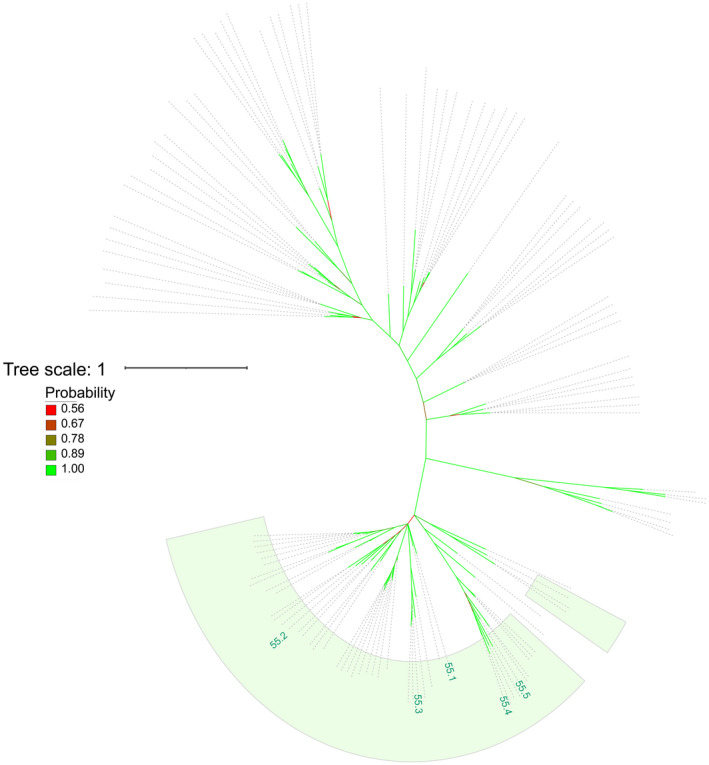
Distribution of the five *helD* genes from *Nonomuraea sp.* ATCC55076. The phylogenetic tree from Figure 1 is shown unannotated apart from boxing the region corresponding to the *Actinobacteria* pale green, and indicating the location of the *Nonomuraea helD* genes: #1 NOA_23645, 772 aa; #2 NOA_16240, 762 aa; #3 NOA_42280, 715 aa; #4 NOA_08745, 660 aa; #5 NOA_48960, 655 aa

A striking observation made in the preliminary phylogenetic analysis of HelD was that some organisms contain more than one *helD* gene (Newing et al., 2020). This preliminary analysis has now been extended and it is clear that the presence of >1 *helD* is common and is found in both Gram‐positive and ‐negative organisms (Figure 3a). Using complete genome sequences, up to 5 genes encoding HelD have been identified (*e*.*g*. *Nonomuraea sp*. ATCC55076 [organism 55]; Figure 3a and Appendix 1; Figure A7), and organisms have been identified with 1, 2, 3, 4, or 5 *helD* genes. Although most contain a single *helD* gene, low G+C Gram‐positives and Gram‐negatives were not found with >3, and high G+C Gram‐positive *Actinobacteria* such as *Streptomyces*, *Nonomuraea*, and *Frankia* were identified with ≥4 *helD* genes. A simple assumption is that these multiple genes are the product of amplification through recombination, and this may well be the root of their original source, but phylogenetic analysis indicates each gene is unique, and organisms with more than one *helD* gene tend to encode both large (~740–850 aa) and small (~680–720 aa) variants. The variation in sequence length is due to differences in the flanking SCA and CA domains (arms) with the core 1A and 2A helicase domains all being of similar size. This suggests the motor function of these proteins is conserved, but the function of large *vs* small HelD variants may differ depending on the size of the SCA and CA arms. The multiple *helD* genes also segregate to Class I, ‐II, or ‐III according to the organism in which they are found; Class I sequences are found in *Firmicutes*, whereas *Actinobacteria* all have Class II sequences (except for the Coriobacterium *Adlercreutzia equolifaciens*, above), and Class III sequences are found in *Deltaproteobacteria*. Of the *Bacteroides*/*Parabacteroides* analyzed to date, all encode only a single Class I *helD* gene.

**FIGURE 7:**
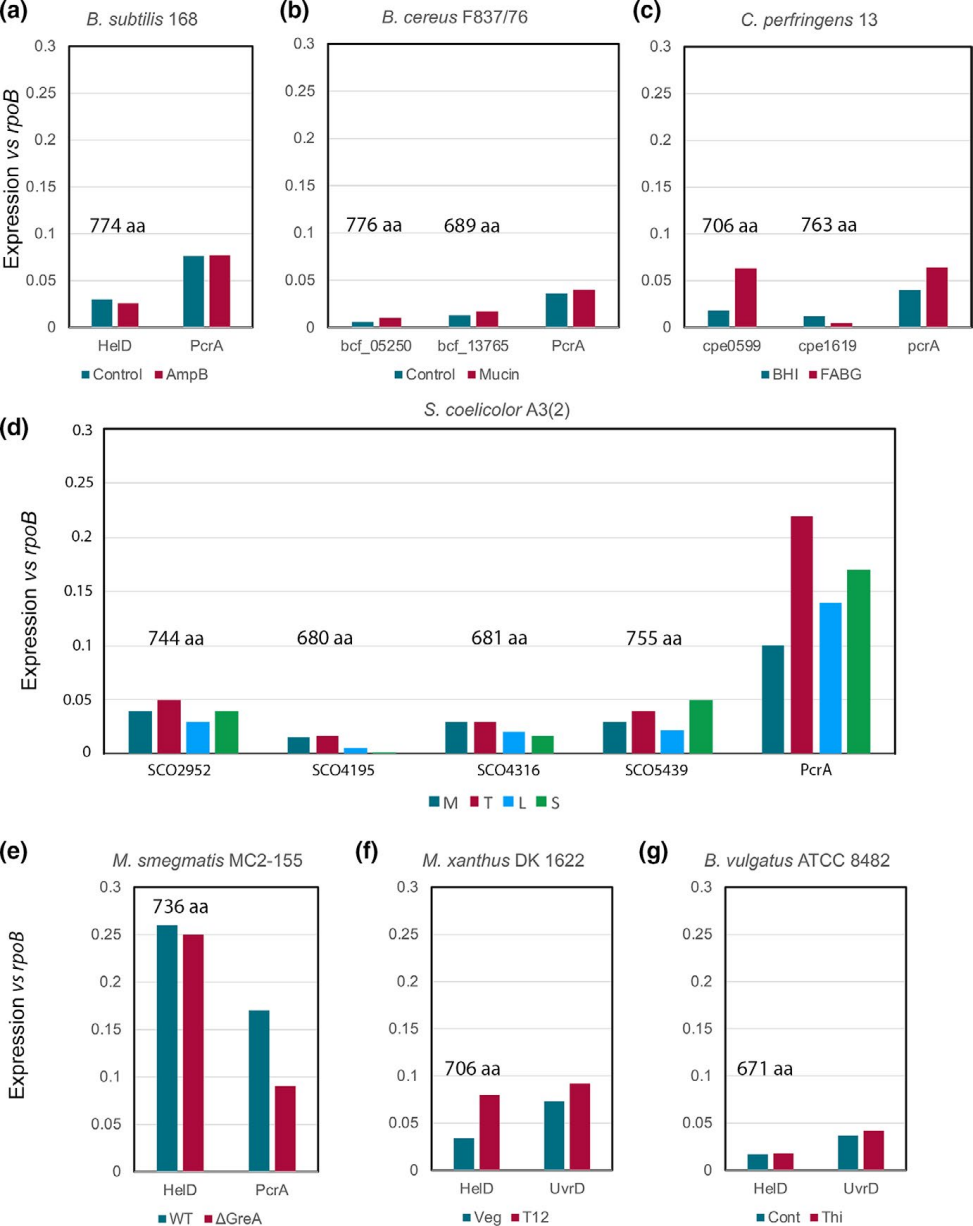
Expression levels of HelD. The relative transcript levels of *helD* and *pcrA*/*uvrD* compared to *rpoB* are shown in panels A–G. Organism names are shown on the top of each plot and gene expression levels are color‐coded according to the keys below the plots. The sizes of the HelD isoforms in amino acids are indicated above the corresponding column in each panel. Details of the sources of the data sets used are provided in the text. A. *B*. *subtilis* 168 data; control teal, amphotericin B (AmpB) treatment red. B. *B*. *cereus* F837/76 data; control teal, mucin treatment red. C. *C*. *perfringens* 13 data; growth in brain heart infusion (BHI) teal, fastidious anaerobic broth +glucose (FABG) red. D. *S*. *coelicolor* A3(2) data; mid‐exponential growth (M) teal, transition phase (T) red, late exponential (L) blue, stationary phase (S) green. E. *M*. *smegmatis* MC2‐155 data; control teal, *greA* deletion strain (ΔGreA) red. F. *M*.* xanthus* DK1622 data; vegetative growth teal, 12 h after initiation of sporulation (T12) red. G. *B*. *vulgatus* ATCC 8482 data; control teal, supplemented with thiamine (Thi) red

Some or all of the additional helD sequences might have represented cryptic genes that are not expressed under any conditions, or that they are differentially expressed during different growth phases or conditions, which might provide clues to potential functions. Transcriptomics data were retrieved from the Sequence Read Archive (SRA) for selected organisms containing 1 or >1 *helD* representative of all three classes of HelD, and expression levels compared relative to *rpoB* (RNAP β subunit) and another housekeeping gene (SF1 helicase *pcrA*/*uvrD*). In all cases, all of the *helD* genes were expressed, often at an approximately similar level to *pcrA*/*uvrD* (Figure 7). The RNA‐seq data of *B*.* subtilis helD* and *pcrA* obtained from experiments by Revilla‐Guarinos et al. (Revilla‐Guarinos et al., 2020) to examine changes in gene expression in a model soil organism on exposure to the antifungal agent amphotericin B produced by *Streptomycetes* closely matched that of the oligonucleotide hybridization transcriptomics data of Nicolas et al. (Nicolas et al., 2012) and showed the level of *helD* expression was not influenced by amphotericin B and was ~3% that of *rpoB* (Figure 7a). This is also consistent with proteomics analysis indicating HelD is present at ~6% the level of RNAP (Delumeau et al., 2011). *B*. *cereus* contains two *helD* genes and the data set from strain F837/76 (Jessberger et al., 2019) grown in the presence and absence of mucin that can influence toxin production shows that both copies (one large, one small variant) are expressed, albeit at low levels, and expression is not significantly affected on exposure to mucin (Figure 7b). *C*. *perfringens* also contains two Class I *helD* genes, labeled CPE_0599 (small; 706 aa) and CPE_1619 (large; 763 aa) in strain 13, and expression levels were determined from datasets of cells grown in brain heart infusion (BHI) and a rich medium developed for the optimal growth of fastidious anaerobes, fastidious anaerobe broth +2% glucose (FABG) medium (Soncini et al., 2020). Both genes were expressed at levels comparable to *helD* in *B*. *subtilis*, and their cognate *prcA*/*uvrD*, although CPE_0599 expression increased ~3‐fold and CPE_1619 expression decreased in FABG medium compared to BHI medium (Figure 6c).

*S*. *coelicolor* A2(3) contains four Class II *helD* genes, two encoding large (SCO_2952 744 aa, and SCO_5439 755 aa) and two encoding small (SCO_4195 680 aa, and SCO_4316 681 aa) variants. Data from a study on growth phase‐dependent changes in gene expression (Jeong et al., 2016) were obtained from the SRA for analysis of *helD* expression and compared with *rpoB* and *pcrA*. All four *helD* genes were expressed with relative levels changing ~2‐fold dependent on the growth phase (Figure 7d). Expression levels were generally highest during mid‐log and transition, and lowest during late and stationary phases, with modest changes between the ratios of expression of the different gene copies at all stages. The RNA‐seq data set for *M*. *smegmatis* comparing changes in gene expression on the deletion of the transcript cleavage factor GreA that is important in rescuing back‐tracked RNAP (Feng et al., 2020) showed that expression of the single *helD* gene was substantially higher than in most other organisms, at about 25% the level of *rpoB* suggesting HelD may be particularly abundant in the *Mycobacteria* (Figure 7e). The expression levels of *helD* were similar in the presence and absence of *greA* indicating each factor acts on stalled transcription complexes independently of each other.

Analysis of RNA‐seq data showed *helD* genes were also expressed in Gram‐negative *M*.* xanthus* and *B*. *vulgatus* (Figure 7f and g), showing that despite the structural differences adjacent to the HelD interaction sites in the β and β’ subunits of RNAP from these organisms, HelDs are expressed and likely able to bind and functionally interact with their cognate RNAPs. The data for *M*.* xanthus* were obtained to examine changes in gene expression during the development of fruiting bodies and spores. It is interesting to note that expression of *helD* in *M*.* xanthus* increases during the development of spores (not to be confused with sporulation in the *Firmicutes*) and may point to a role in the storage of inactive RNAP during dormancy as has been proposed for *B*. *subtilis* HelD (Pei et al., 2020). The study in *B*. *vulgatus* was designed to investigate the effect on gene expression of exogenous thiamine that may be important in niche establishment in the gut. Therefore, in most/all organisms that contain *helD* gene(s), it/they are expressed. The reason why one organism contains a single gene and closely related species contain more than one (*e*.*g*. *B*. *subtilis* and *B*. *cereus*, Figure 6a and b) is currently not clear, but the expression data would suggest that each isoform has a functional role to play in the cell, and there is not a significant difference in the expression of large *vs* small *helD* variants.

## 4 CONCLUSIONS

**FIGURE A8:**
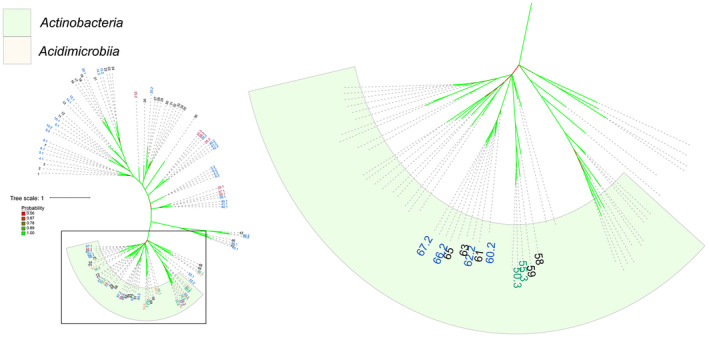
The *helR* group of *helD* variants that (potentially) confer rifampicin resistance. The phylogenetic tree from Figure 1 is shown on the left side with the region boxed expanded on the right side. Bacterial classes are colored, species numbered, the number of *helD* genes colored as in Figure 1. Only the (potential) *helR* variants are shown: **50** *Streptomyces venezuelae* (#3 SVEN_6029). **55** *Nonomuraea* sp. ATCC55076 (#3 NOA_42280, 715 aa). **58** *Microbacterium sp*. PAMC 28756 (mip_00070, 717aa). **59** *Mirobacterium hominis* SJTG1 (mhos_01135, 744aa). **60** *Nocardia farcinica* IFM10152 (#2 NFA_44160, 726aa). **61** *Mycobacterium smegmatis* MC2 155 (MSMEG_2174, 736aa). **62** *Rhodococcus* sp. 008 (#2 rhod_09075, 731aa). **63** *Mycobacterium sp*. JS623 (Mycsm_03949, 732aa). **65** *Mycobacteroides abscessus* ATCC 19977 (MAB_3189c, 753aa). **66** *Rhodococcus equi* 103S (#2 REQ_15310, 739aa). **67** *Nocardia asteroides* NCTC11293 (#2 nad_04408, 735aa). One *helD* gene, black; two, blue; five, green

In this work, we have examined the phylogenetic distribution and classification of the transcription recycling factor HelD in detail and have identified a new class restricted to the *Deltaproteobacteria*. In addition, it appears *helD* genes have been acquired by horizontal transfer on at least three occasions; *Bacteroides* have acquired *helD* from the *Clostridiales*, whereas the *Coriobacteria* have acquired it from the *Lactobacilli* and *Clostridiales*. The gut microbiome is known as an environment conducive to horizontal gene transfer, especially with respect to the distribution of antibiotic resistance genes (McInnes et al., 2020), and given that *Bacteroides*, *Lactobacilli*, *Clostridiales*, and *Coriobacteria* are all common in the gut microbiome, it appears *A*. *equolifaciens* has acquired *helD* genes from gut microorganisms on two separate occasions. Indeed, an unusual feature of *helD* genes is that many organisms contain multiple paralogues and that all versions are expressed. Why some organisms have a single gene for *helD* while a closely related species has multiple expressed copies is unclear, and this will make a fascinating avenue for future research. It is interesting to note that actinobacteria, such as *Streptomyces*, *Frankia*, and *Nonomuraea* (numbers 50, 51, 54, and 55; Figure 3) that are known producers of valuable bioactive compounds used as antibiotics and anti‐cancer drugs contained the largest number of *helD* genes (4–5). The 5 helD genes in *Nonomuraea* (number 55, Figure 3), which is a known producer of DNA‐intercalating agents (Sungthong & Nakaew, 2015) may be involved in genome maintenance through recycling stalled transcription complexes during the production of these compounds. *Nonomuraea* and other *Actinomycetales* sometimes have a second *rpoB* gene that confers resistance of RNAP to compounds such as rifampicin and sorangicin that is induced by stress and is associated with the production of secondary metabolites (D'Argenio et al., 2016). The combination of multiple HelD isoforms with drug‐resistant RNAP may be important in this proposed genome maintenance activity. In some organisms, such as *M*. *abcessus* and *S*.* venezuaelae helD* expression is induced in the presence of the antibiotic rifampicin, conferring resistance, and this is associated with the presence of a DNA sequence called the Rifamycin Associated Element (RAE) found upstream of the gene (Hurst‐Hess et al., 2021; Surette et al., 2021). It is proposed that the tip of the PCh loop can physically remove rifampicin bound to the RNAP β subunit in a pocket close to the active site. In *S*. *venezuelae* (organism #50, Figure 3) that has five *helD* genes, only one (SVEN_6029, #50.3) is induced in the presence of rifampicin and has an upstream RAE (Surette et al., 2021). It is interesting to note that despite encoding a rifampicin‐resistant RNAP β subunit, *Nonomuraea* also has an RAE located directly upstream of *helD* NOA_42280 (#55.3; Appendix 1; Figures A7 and A8).

Investigation of the distribution of *helD* genes with upstream RAEs revealed they were clustered to two sub‐branches of the *Actinobacteria* (Appendix 1; Figure A8) that may be considered the HelR grouping based on the nomenclature of these proteins by (Hurst‐Hess et al., 2021; Surette et al., 2021). It should be noted that clearly identifiable RAEs could not be found upstream of all the genes in the HelR group, including for *Frankia alni*, *Nocardia brasiliensis*, or *Mycolicibacterium phlei* (54.2, 56.2, and 64, respectively; Figure 3 and Appendix 1 Figure A2). Rifampicin has also been observed to induce *helD* expression in the low G+C Gram‐positive *B*. *subtilis*, but this induction does not confer resistance to the drug (Hutter et al., 2004). Nevertheless, the ability of naturally produced antibiotics to induce the expression of *helD* genes suggests HelD proteins have a potentially important role in preserving genome integrity and gene expression in the bacteria in which they are found.

An additional area of future research should include functional and structural studies of HelD from Gram‐negative bacteria, as due to the location of lineage‐specific inserts in the β and β’ subunits of RNAP in Gram‐negatives it was assumed HelD‐like proteins would bind poorly or be sterically inhibited from binding. HelD proteins represent a new class of motor enzymes involved in transcription complex recycling that are widely distributed in bacteria that make an important contribution to our understanding of the multiple different mechanisms used to resolve potentially lethal stalled transcription complexes.

Finally, it is important that genome annotation databases are updated as *helD* genes are often classified as *pcrA*, *uvrD*, or helicase IV‐ATPase. Correct annotation of *helD* genes will enable a more detailed understanding of the distribution, evolution, and function of this fascinating new category of transcription factors.

## 5 ETHICS STATEMENT

None required.

## CONFLICT OF INTEREST

None declared.

## AUTHOR CONTRIBUTIONS

**Joachim S. Larsen:** Formal analysis (supporting); Methodology (supporting); Software (supporting); Writing‐review & editing (supporting). **Michael Miller:** Formal analysis (supporting); Writing‐review & editing (supporting). **Aaron J. Oakley:** Formal analysis (supporting); Funding acquisition (equal); Writing‐review & editing (supporting). **Nicholas E. Dixon:** Formal analysis (supporting); Funding acquisition (equal); Writing‐review & editing (supporting). **Peter Lewis:** Conceptualization (lead); Data curation (lead); Formal analysis (lead); Funding acquisition (equal); Investigation (lead); Methodology (lead); Project administration (lead); Resources (lead); Supervision (lead); Writing‐original draft (lead); Writing‐review & editing (lead).

## Figures and Tables

**TABLE A1:**
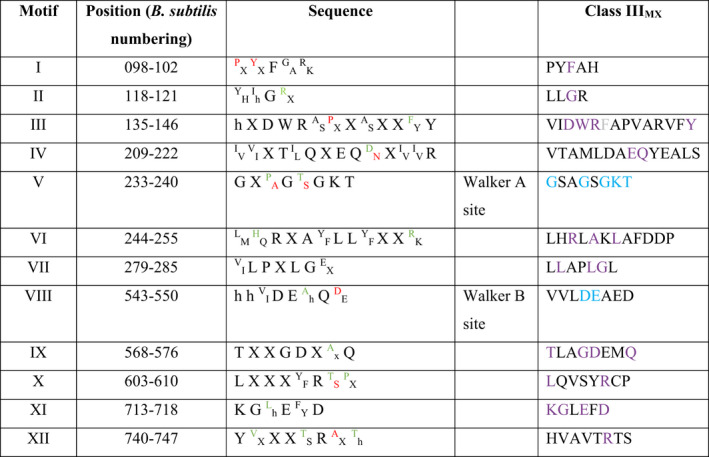
comparison of conserved class I and II HelD motifs with those from class III M. xanthus HelD

## Data Availability

All data are provided in full in the results section of this paper and all sequences used are available from the NCBI at https://www.ncbi.nlm.nih.gov

## References

[mbo31251-bib-0001] Adelman, K. , & Lis, J. T. (2012). Promoter‐proximal pausing of RNA polymerase II: Emerging roles in metazoans. Nature Reviews Genetics, 13, 720–731. 10.1038/nrg3293 PMC3552498 22986266

[mbo31251-bib-0002] Clavel, T. , Lepage, P. , & Charrier, C. (2014). The family coriobacteriaceae. In: E. Rosenberg , E. F. Delong , S. Lory , E. Stackebrandt , & F. Thompson (Eds.), The prokaryotes (pp. 201–238). Springer.

[mbo31251-bib-0003] D'Argenio, V. , Petrillo, M. , Pasanisi, D. , Pagliarulo, C. , Colicchio, R. , Tala, A. , de Biase, M. S. , Zanfardino, M. , Scolamiero, E. , Pagliuca, C. , Gaballo, A. , Cicatiello, A. G. , Cantiello, P. , Postiglione, I. , Naso, B. , Boccia, A. , Durante, M. , Cozzuto, L. , Salvatore, P. , … Alifano, P. (2016). The complete 12 Mb genome and transcriptome of Nonomuraea gerenzanensis with new insights into its duplicated "magic" RNA polymerase. Scientific Reports, 6, 18. 28442708 10.1038/s41598-016-0025-0 PMC5431353

[mbo31251-bib-0004] Darriba, D. , Taboada, G. L. , Doallo, R. , & Posada, D. (2011). ProtTest 3: Fast selection of best‐fit models of protein evolution. Bioinformatics, 27, 1164–1165. 21335321 10.1093/bioinformatics/btr088 PMC5215816

[mbo31251-bib-0005] Delumeau, O. , Lecointe, F. , Muntel, J. , Guillot, A. , Guedon, E. , Monnet, V. , Hecker, M. , Becher, D. , Polard, P. , & Noirot, P. (2011). The dynamic protein partnership of RNA polymerase in *Bacillus subtilis* . Proteomics, 11, 2992–3001. 21710567 10.1002/pmic.201000790

[mbo31251-bib-0006] Epshtein, V. , Kamarthapu, V. , McGary, K. , Svetlov, V. , Ueberheide, B. , Proshkin, S. , Mironov, A. , & Nudler, E. (2014). UvrD facilitates DNA repair by pulling RNA polymerase backwards. Nature, 505, 372–377. 24402227 10.1038/nature12928 PMC4471481

[mbo31251-bib-0007] Feng, S. , Liu, Y. , Liang, W. , El‐Sayed Ahmed, M. A. E. , Zhao, Z. , Shen, C. , Roberts, A. P. , Liang, L. , Liao, L. , Zhong, Z. , Guo, Z. , Yang, Y. , Wen, X. , Chen, H. , & Tian, G. B. (2020). Involvement of transcription elongation factor GreA in Mycobacterium viability, antibiotic susceptibility, and intracellular fitness. Frontiers in Microbiology, 11, 413. 32265867 10.3389/fmicb.2020.00413 PMC7104715

[mbo31251-bib-0008] Geer, L. Y. , Domrachev, M. , Lipman, D. J. , & Bryant, S. H. (2002). CDART: Protein homology by domain architecture. Genome Research, 12, 1619–1623. 12368255 10.1101/gr.278202 PMC187533

[mbo31251-bib-0009] Ghodke, H. , Ho, H. N. , & van Oijen, A. M. (2020). Single‐molecule live‐cell imaging visualizes parallel pathways of prokaryotic nucleotide excision repair. Nature Communications, 11, 1477. 10.1038/s41467-020-15179-y PMC7083872 32198385

[mbo31251-bib-0010] Gupta, M. K. , Guy, C. P. , Yeeles, J. T. , Atkinson, J. , Bell, H. , Lloyd, R. G. , Marians, K. J. , & McGlynn, P. (2013). Protein‐DNA complexes are the primary sources of replication fork pausing in *Escherichia coli* . Proceedings National Academy Sciences of the United States of America, 110, 7252–7257. 10.1073/pnas.1303890110 PMC3645559 23589869

[mbo31251-bib-0011] Hawkins, M. , Dimude, J. U. , Howard, J. A. L. , Smith, A. J. , Dillingham, M. S. , Savery, N. J. , Rudolph, C. J. , & McGlynn, P. (2019). Direct removal of RNA polymerase barriers to replication by accessory replicative helicases. Nucleic Acids Research, 47, 5100–5113. 30869136 10.1093/nar/gkz170 PMC6547429

[mbo31251-bib-0012] Ho, H. N. , van Oijen, A. M. , & Ghodke, H. (2018). The transcription‐repair coupling factor Mfd associates with RNA polymerase in the absence of exogenous damage. Nature Communications, 9, 1570. 10.1038/s41467-018-03790-z PMC5910403 29679003

[mbo31251-bib-0013] Huelsenbeck, J. P. , & Ronquist, F. (2001). MRBAYES: Bayesian inference of phylogenetic trees. Bioinformatics, 17, 754–755. 11524383 10.1093/bioinformatics/17.8.754

[mbo31251-bib-0014] Hurst‐Hess, K. R. , Saxena, A. , & Ghosh, P. (2021). Mycobacterium abscessus HelR interacts with RNA Polymerase to confer intrinsic rifamycin resistance. *bioRxiv*, 2021.05.10.443476. 10.1016/j.molcel.2022.06.034 PMC9444957 35905736

[mbo31251-bib-0015] Hutter, B. , Fischer, C. , Jacobi, A. , Schaab, C. , & Loferer, H. (2004). Panel of *Bacillus subtilis* reporter strains indicative of various modes of action. Antimicrobial Agents and Chemotherapy, 48, 2588–2594. 15215113 10.1128/AAC.48.7.2588-2594.2004 PMC434206

[mbo31251-bib-0016] Jeong, Y. , Kim, J. N. , Kim, M. W. , Bucca, G. , Cho, S. , Yoon, Y. J. , Kim, B. G. , Roe, J. H. , Kim, S. C. , Smith, C. P. , & Cho, B. K. (2016). The dynamic transcriptional and translational landscape of the model antibiotic producer *Streptomyces coelicolor* A3(2). Nature Communications, 7, 11605. 10.1038/ncomms11605 PMC4895711 27251447

[mbo31251-bib-0017] Jessberger, N. , Dietrich, R. , Mohr, A. K. , da Riol, C. , & Martlbauer, E. (2019). Porcine gastric mucin triggers toxin production of enteropathogenic *Bacillus cere*us. Infection and Immunity, 87, e00765‐18. 30745328 10.1128/IAI.00765-18 PMC6434126

[mbo31251-bib-0018] Jumper, J. , Evans, R. , Pritzel, A. , Green, T. , Figurnov, M. , Ronneberger, O. , Tunyasuvunakool, K. , Bates, R. , Zidek, A. , Potapenko, A. , Bridgland, A. , Meyer, C. , Kohl, S. A. A. , Ballard, A. J. , Cowie, A. , Romera‐Paredes, B. , Nikolov, S. , Jain, R. , Adler, J. , … Hassabis, D. (2021). Highly accurate protein structure prediction with AlphaFold. Nature, 596(7873), 583–589. 10.1038/s41586-021-03819-2 34265844 PMC8371605

[mbo31251-bib-0019] Kang, J. Y. , Llewellyn, E. , Chen, J. , Olinares, P. D. B. , Brewer, J. , Chait, B. T. , Campbell, E. A. , & Darst, S. A. (2021). Structural basis for transcription complex disruption by the Mfd translocase. Elife, 10, e62117. 33480355 10.7554/eLife.62117 PMC7864632

[mbo31251-bib-0020] Kang, J. Y. , Olinares, P. D. , Chen, J. , Campbell, E. A. , Mustaev, A. , Chait, B. T. , Gottesman, M. E. , & Darst, S. A. (2017). Structural basis of transcription arrest by coliphage HK022 Nun in an *Escherichia coli* RNA polymerase elongation complex. Elife, 6, e25478. 28318486 10.7554/eLife.25478 PMC5386594

[mbo31251-bib-0021] Katoh, K. , Misawa, K. , Kuma, K. , & Miyata, T. (2002). MAFFT: A novel method for rapid multiple sequence alignment based on fast Fourier transform. Nucleic Acids Research, 30, 3059–3066. 12136088 10.1093/nar/gkf436 PMC135756

[mbo31251-bib-0022] Katoh, K. , Rozewicki, J. , & Yamada, K. D. (2019). MAFFT online service: Multiple sequence alignment, interactive sequence choice and visualization. Briefings in Bioinformatics, 20, 1160–1166. 28968734 10.1093/bib/bbx108 PMC6781576

[mbo31251-bib-0023] Keller, A. N. , Yang, X. , Wiedermannova, J. , Delumeau, O. , Krasny, L. , & Lewis, P. J. (2014). epsilon, a new subunit of RNA polymerase found in gram‐positive bacteria. Journal of Bacteriology, 196, 3622–3632. 25092033 10.1128/JB.02020-14 PMC4187704

[mbo31251-bib-0024] Kouba, T. , Koval, T. , Sudzinova, P. , Pospisil, J. , Brezovska, B. , Hnilicova, J. , Sanderova, H. , Janouskova, M. , Sikova, M. , Halada, P. , Sykora, M. , Barvik, I. , Novacek, J. , Trundova, M. , Duskova, J. , Skalova, T. , Chon, U. , Murakami, K. S. , Dohnalek, J. , & Krasny, L. (2020). Mycobacterial HelD is a nucleic acids‐clearing factor for RNA polymerase. Nature Communications, 11, 6419. 10.1038/s41467-020-20158-4 PMC7749160 33339823

[mbo31251-bib-0025] Koval, T. , Sudzinova, P. , Perhacova, T. , Trundova, M. , Skalova, T. , Fejfarova, K. , Sanderova, H. , Krasny, L. , Duskova, J. , & Dohnalek, J. (2019). Domain structure of HelD, an interaction partner of Bacillus subtilis RNA polymerase. FEBS Letters, 593, 996–1005. 30972737 10.1002/1873-3468.13385

[mbo31251-bib-0026] Kumar, S. , Stecher, G. , Li, M. , Knyaz, C. , & Tamura, K. (2018). MEGA X: Molecular evolutionary genetics analysis across computing platforms. Molecular Biology and Evolution, 35, 1547–1549. 29722887 10.1093/molbev/msy096 PMC5967553

[mbo31251-bib-0027] Lane, W. J. , & Darst, S. A. (2010a). Molecular evolution of multisubunit RNA polymerases: Sequence analysis. Journal of Molecular Biology, 395, 671–685. 19895820 10.1016/j.jmb.2009.10.062 PMC2813377

[mbo31251-bib-0028] Lane, W. J. , & Darst, S. A. (2010b). Molecular evolution of multisubunit RNA polymerases: Structural analysis. Journal of Molecular Biology, 395, 686–704. 19895816 10.1016/j.jmb.2009.10.063 PMC2813324

[mbo31251-bib-0029] Le, T. T. , Yang, Y. , Tan, C. , Suhanovsky, M. M. , Fulbright, R. M. , Inman, J. T. , Li, M. , Lee, J. , Perelman, S. , Roberts, J. W. , Deaconescu, A. M. , & Wang, M. D. (2018). Mfd dynamically regulates transcription via a release and catch‐up mechanism. Cell, 172, 344–357 e15. 29224782 10.1016/j.cell.2017.11.017 PMC5766421

[mbo31251-bib-0030] Letunic, I. , & Bork, P. (2019). Interactive Tree Of Life (iTOL) v4: Recent updates and new developments. Nucleic Acids Research, 47, W256–W259. 30931475 10.1093/nar/gkz239 PMC6602468

[mbo31251-bib-0031] Liu, B. , Zuo, Y. , & Steitz, T. A. (2015). Structural basis for transcription reactivation by RapA. Proceedings National Academy Sciences of the United States of America, 112, 2006–2010. 10.1073/pnas.1417152112 PMC4343176 25646438

[mbo31251-bib-0032] Lopetuso, L. R. , Scaldaferri, F. , Petito, V. , & Gasbarrini, A. (2013). Commensal Clostridia: Leading players in the maintenance of gut homeostasis. Gut Pathogology, 5, 23. 10.1186/1757-4749-5-23 PMC3751348 23941657

[mbo31251-bib-0033] Lozupone, C. A. , Stombaugh, J. I. , Gordon, J. I. , Jansson, J. K. , & Knight, R. (2012). Diversity, stability and resilience of the human gut microbiota. Nature, 489, 220–230. 22972295 10.1038/nature11550 PMC3577372

[mbo31251-bib-0034] McInnes, R. S. , McCallum, G. E. , Lamberte, L. E. , & van Schaik, W. (2020). Horizontal transfer of antibiotic resistance genes in the human gut microbiome. Current Opinion in Microbiology, 53, 35–43. 32143027 10.1016/j.mib.2020.02.002

[mbo31251-bib-0035] Mendler, K. , Chen, H. , Parks, D. H. , Lobb, B. , Hug, L. A. , & Doxey, A. C. (2019). AnnoTree: visualization and exploration of a functionally annotated microbial tree of life. Nucleic Acids Research, 47, 4442–4448. 31081040 10.1093/nar/gkz246 PMC6511854

[mbo31251-bib-0036] Milani, C. , Duranti, S. , Bottacini, F. , Casey, E. , Turroni, F. , Mahony, J. , Belzer, C. , Delgado Palacio, S. , Arboleya Montes, S. , Mancabelli, L. , Lugli, G. A. , Rodriguez, J. M. , Bode, L. , De Vos, W. , Gueimonde, M. , Margolles, A. , van Sinderen, D. , & Ventura, M. (2017). The first microbial colonizers of the human gut: Composition, activities, and health implications of the infant gut microbiota. Microbiology Molecular Biology Reviews, 81, e00036‐17. 10.1128/MMBR.00036-17 PMC5706746 29118049

[mbo31251-bib-0037] Miller, M. , Oakley, A. J. & Lewis, P. J. (2021). RNA polymerases from Low G+C Gram Positive Bacteria. bioRxiv 2021.06.06.447298. 10.1080/21541264.2021.1964328 PMC8632073 34403307

[mbo31251-bib-0038] Newing, T. P. , Oakley, A. J. , Miller, M. , Dawson, C. J. , Brown, S. H. J. , Bouwer, J. C. , Tolun, G. , & Lewis, P. J. (2020). Molecular basis for RNA polymerase‐dependent transcription complex recycling by the helicase‐like motor protein HelD. Nature Communications, 11, 6420. 10.1038/s41467-020-20157-5 PMC7749167 33339820

[mbo31251-bib-0039] Nicolas, P. , Mader, U. , Dervyn, E. , Rochat, T. , Leduc, A. , Pigeonneau, N. , Bidnenko, E. , Marchadier, E. , Hoebeke, M. , Aymerich, S. , Becher, D. , Bisicchia, P. , Botella, E. , Delumeau, O. , Doherty, G. , Denham, E. L. , Fogg, M. J. , Fromion, V. , Goelzer, A. , … Noirot, P. (2012). Condition‐dependent transcriptome reveals high‐level regulatory architecture in *Bacillus subtilis* . Science, 335, 1103–1106. 22383849 10.1126/science.1206848

[mbo31251-bib-0040] Pei, H. H. , Hilal, T. , Chen, Z. A. , Huang, Y. H. , Gao, Y. , Said, N. , Loll, B. , Rappsilber, J. , Belogurov, G. A. , Artsimovitch, I. , & Wahl, M. C. (2020). The delta subunit and NTPase HelD institute a two‐pronged mechanism for RNA polymerase recycling. Nature Communications, 11, 6418. 10.1038/s41467-020-20159-3 PMC7749165 33339827

[mbo31251-bib-0041] Pettersen, E. F. , Goddard, T. D. , Huang, C. C. , Meng, E. C. , Couch, G. S. , Croll, T. I. , Morris, J. H. , & Ferrin, T. E. (2021). UCSF ChimeraX: Structure visualization for researchers, educators, and developers. Protein Science, 30(1), 70–82. 10.1002/pro.3943 32881101 PMC7737788

[mbo31251-bib-0042] Pomerantz, R. T. , & O'Donnell, M. (2008). The replisome uses mRNA as a primer after colliding with RNA polymerase. Nature, 456, 762–766. 19020502 10.1038/nature07527 PMC2605185

[mbo31251-bib-0043] Pomerantz, R. T. , & O'Donnell, M. (2010). Direct restart of a replication fork stalled by a head‐on RNA polymerase. Science, 327, 590–592. 20110508 10.1126/science.1179595 PMC2861996

[mbo31251-bib-0044] Poyet, M. , Groussin, M. , Gibbons, S. M. , Avila‐Pacheco, J. , Jiang, X. , Kearney, S. M. , Perrotta, A. R. , Berdy, B. , Zhao, S. , Lieberman, T. D. , Swanson, P. K. , Smith, M. , Roesemann, S. , Alexander, J. E. , Rich, S. A. , Livny, J. , Vlamakis, H. , Clish, C. , Bullock, K. , … Alm, E. J. (2019). A library of human gut bacterial isolates paired with longitudinal multiomics data enables mechanistic microbiome research. Nature Medicine, 25, 1442–1452. 10.1038/s41591-019-0559-3 31477907

[mbo31251-bib-0045] Ragheb, M. N. , Merrikh, C. , Browning, K. , & Merrikh, H. (2021). Mfd regulates RNA polymerase association with hard‐to‐transcribe regions in vivo, especially those with structured RNAs. Proceedings of the National Academy of Sciences, 118(1), e2008498118. 10.1073/pnas.2008498118 PMC7817204 33443179

[mbo31251-bib-0046] Revilla‐Guarinos, A. , Durr, F. , Popp, P. F. , Doring, M. , & Mascher, T. (2020). Amphotericin B specifically induces the two‐component system LnrJK: Development of a novel whole‐cell biosensor for the detection of amphotericin‐like polyenes. Frontiers in Microbiology, 11, 2022. 32973732 10.3389/fmicb.2020.02022 PMC7472640

[mbo31251-bib-0047] Rocha, E. P. C. (2004). The replication‐related organization of bacterial genomes. Microbiology, 150, 1609–1627. 15184548 10.1099/mic.0.26974-0

[mbo31251-bib-0048] Ronquist, F. , Teslenko, M. , van der Mark, P. , Ayres, D. L. , Darling, A. , Hohna, S. , Larget, B. , Liu, L. , Suchard, M. A. , & Huelsenbeck, J. P. (2012). MrBayes 3.2: efficient Bayesian phylogenetic inference and model choice across a large model space. Systematic Biology, 61, 539–542. 10.1093/sysbio/sys029 22357727 PMC3329765

[mbo31251-bib-0049] Saba, J. , Chua, X. Y. , Mishanina, T. V. , Nayak, D. , Windgassen, T. A. , Mooney, R. A. , & Landick, R. (2019). The elemental mechanism of transcriptional pausing. eLife, 8, e40981. 10.7554/elife.40981 30618376 PMC6336406

[mbo31251-bib-0050] Shi, J. , Wen, A. , Zhao, M. , Jin, S. , You, L. , Shi, Y. , Dong, S. , Hua, X. , Zhang, Y. , & Feng, Y. (2020). Structural basis of Mfd‐dependent transcription termination. Nucleic Acids Research, 48, 11762–11772. 33068413 10.1093/nar/gkaa904 PMC7672476

[mbo31251-bib-0051] Sikova, M. , Wiedermannova, J. , Prevorovsky, M. , Barvik, I. , Sudzinova, P. , Kofronova, O. , Benada, O. , Sanderova, H. , Condon, C. , & Krasny, L. (2020). The torpedo effect in *Bacillus subtilis*: RNase J1 resolves stalled transcription complexes. EMBO Journal, 39, e102500. 10.15252/embj.2019102500 PMC6996504 31840842

[mbo31251-bib-0052] Soncini, S. R. , Hartman, A. H. , Gallagher, T. M. , Camper, G. J. , Jensen, R. V. , & Melville, S. B. (2020). Changes in the expression of genes encoding type IV pili‐associated proteins are seen when *Clostridium perfringens* is grown in liquid or on surfaces. BMC Genomics, 21, 45. 10.1186/s12864-020-6453-z 31937237 PMC6958937

[mbo31251-bib-0053] Stamatakis, A. (2006). RAxML‐VI‐HPC: maximum likelihood‐based phylogenetic analyses with thousands of taxa and mixed models. Bioinformatics, 22, 2688–2690. 10.1093/bioinformatics/btl446 16928733

[mbo31251-bib-0054] Sungthong, R. , & Nakaew, N. (2015). The genus Nonomuraea: A review of a rare actinomycete taxon for novel metabolites. Journal of Basic Microbiology, 55, 554–565. 24633812 10.1002/jobm.201300691

[mbo31251-bib-0055] Surette, M. D. , Waglechner, N. , Koteva, K. , & Wright, G. D. (2021). HelR is a helicase‐like protein that protects RNA polymerase from rifamycin antibiotics. *bioRxiv* 2021.05.10.443488. 10.1016/j.molcel.2022.06.019 35907401

[mbo31251-bib-0056] Takahashi, T. , Satoh, I. , & Kikuchi, N. (1999). Phylogenetic relationships of 38 taxa of the genus *Staphylococcus* based on 16S rRNA gene sequence analysis. International Journal of Systematic Bacteriology, 49(Pt 2), 725–728. 10319495 10.1099/00207713-49-2-725

[mbo31251-bib-0057] Urrutia‐Irazabal, I. , Ault, J. R. , Sobott, F. , Savery, N. J. , & Dillingham, M. S. (2021). Analysis of the PcrA‐RNA polymerase complex reveals a helicase interaction motif and a role for PcrA/UvrD helicase in the suppression of R‐loops. eLife, 10, e68829. 10.7554/elife.68829 34279225 PMC8318588

[mbo31251-bib-0058] Waterhouse, A. , Bertoni, M. , Bienert, S. , Studer, G. , Tauriello, G. , Gumienny, R. , Heer, F. T. , de Beer, T. A. P. , Rempfer, C. , Bordoli, L. , Lepore, R. , & Schwede, T. (2018). SWISS‐MODEL: Homology modelling of protein structures and complexes. Nucleic Acids Research, 46, W296–W303. 29788355 10.1093/nar/gky427 PMC6030848

[mbo31251-bib-0059] Weiss, A. , & Shaw, L. N. (2015). Small things considered: The small accessory subunits of RNA polymerase in Gram‐positive bacteria. FEMS Microbiology Reviews, 39, 541–554. 25878038 10.1093/femsre/fuv005 PMC4498139

[mbo31251-bib-0060] Westblade, L. F. , Campbell, E. A. , Pukhrambam, C. , Padovan, J. C. , Nickels, B. E. , Lamour, V. , & Darst, S. A. (2010). Structural basis for the bacterial transcription‐repair coupling factor/RNA polymerase interaction. Nucleic Acids Research, 38, 8357–8369. 20702425 10.1093/nar/gkq692 PMC3001067

[mbo31251-bib-0061] Wiedermannova, J. , Sudzinova, P. , Koval, T. , Rabatinova, A. , Sanderova, H. , Ramaniuk, O. , Rittich, S. , Dohnalek, J. , Fu, Z. , Halada, P. , Lewis, P. , & Krasny, L. (2014). Characterization of HelD, an interacting partner of RNA polymerase from *Bacillus subtilis* . Nucleic Acids Research, 42, 5151–5163. 24520113 10.1093/nar/gku113 PMC4005671

[mbo31251-bib-0062] Wood, E. R. , & Matson, S. W. (1987). Purification and characterization of a new DNA‐dependent ATPase with helicase activity from *Escherichia coli* . Journal of Biological Chemistry, 262, 15269–15276. 2822720

[mbo31251-bib-0063] Yang, J. , Yan, R. , Roy, A. , Xu, D. , Poisson, J. , & Zhang, Y. (2015). The I‐TASSER Suite: Protein structure and function prediction. Nature Methods, 12, 7–8. 25549265 10.1038/nmeth.3213 PMC4428668

